# The Effect of Sulphur Atom on the Structure of Biomolecule 2-Thiocytosine in the Gas-Phase, Solid-State, and Hydrated Forms and in DNA–DNA Microhelices as Compared to Canonical Ones

**DOI:** 10.3390/molecules30030559

**Published:** 2025-01-26

**Authors:** Mauricio Alcolea Palafox, Valentin Alba Aparicio, Sergio Toninelli Rodriguez, Josefa Isasi Marín, Jitendra Kumar Vats, Vinod Kumar Rastogi

**Affiliations:** 1 Departamento de Química-Física, Facultad de Ciencias Químicas, Universidad Complutense, 28040 Madrid, Spain; valalba@ucm.es (V.A.A.); sergtoni@ucm.es (S.T.R.); 2 Departamento de Química Inorgánica, Facultad de Ciencias Químicas, Universidad Complutense, 28040 Madrid, Spain; isasi@ucm.es; 3 P G Department of Physics, RJC, Jai Prakash University, Chapra 841301, India; drjkvats@gmail.com; 4 Indian Spectroscopy Society, KC-68/1, Old Kavinagar, Ghaziabad 201002, India; v_krastogi@rediffmail.com

**Keywords:** WC pair, 2-thiocytosine, thiopyrimidine, vibrational analysis, microhelix, DNA

## Abstract

This study is focused on the effects of the sulphur atom in position 2 of the cytosine molecule, 2-thiocytosine (2TC), on the molecular structural parameters in the isolated state, as well as in the hydration, solid state arrangement, Watson–Crick pairs, and DNA–DNA microhelices, as compared to the canonical form. The main six tautomers were optimised at the MP2 and CCSD levels, and the sulphur atom does not show any effect on the stability trend of cytosine. The energy difference between **T2b** and **T2a** tautomers is twice as low in 2TC (1.15 kJ/mol) than in cytosine (2.69 kJ/mol). The IR and laser Raman spectra of 2TC were accurately assigned using DFT computations and solid-state simulations of the crystal unit cell through several tetramer forms. The results notably improve those previously published by other authors. The effect of explicit water molecules surrounding 2TC up to 30, corresponding to the first and second hydration shells, on geometries and tautomerism was analysed. The Watson–Crick base pairs’ stability (ΔE^CP^ = −97.458 kJ/mol) was found to be less than with cytosine (−105.930 kJ/mol). The calculated dipole moment was also lower (4.205 D) than with cytosine (5.793 D). The effect of 2TC on the 5′-dG-dC-dG-3′ and 5′-dA-dC-dA-3′ DNA–DNA optimised microhelices was evaluated through their calculated helical parameters, which indicates a clear deformation of the helix formation. The radius (R) with 2TC appears considerably shorter (6.200 Å) in the 5′-dA-dC-dA-3′ microhelix than that with cytosine (7.050 Å). Because of the special characteristics of the 2TC molecule, it can be used as an anticancer drug.

## 1. Introduction

Nucleic acids consisting of two types of nitrogen bases called purines and pyrimidines are the most fascinating molecules found in living organisms. One of the DNA constituents is cytosine nucleobase, and therefore, this molecule and its derivatives are of great importance and have diverse biological potential [1,2,3]. However, the detection of sulphur in natural tRNA in 1965 [4,5] led to many studies on thio-substituted compounds [6,7]. Substituting the O2 oxygen of cytosine molecule with a sulphur atom leads to 2-thiocytosine (2TC) which is present in the anticodon loop of the bacterial t-RNA [8,9,10]. Many thio-derivatives of nucleobases are of special interest not only owing to their effect on nucleic acid structures being unknown but also because they exhibit noticeable pharmacological activities. Therefore, they are important compounds in various metabolic processes [11] and are commonly used as antitumor drugs [12] in clinical treatments. The properties of purine and pyrimidine bases are determined by their hydrogen and π-bonding systems [13], and interactions involving the sulphur substituent may contribute to the unusual physical and biological properties of nucleic acids containing it. For this reason, the effect of substituting sulphur instead of oxygen on the molecular structure, chemical properties, and biological activities of pyrimidines was studied some time ago [14].

As with other thio-pyrimidines, 2TC may have played a key role in the prebiotic synthesis of nucleic acids [15] and in the first RNAs in the early state of the Earth, the so-called HCN scenario of life origin [16]. 2TC is adsorbed on gold nanoparticle surfaces [17] and forms gold complexes that show potent inhibition of thioredoxin reductase activity [18]. It also forms metal complexes with several divalent metals, such as Ti [19], where the C-S group appears as a coordination site through direct interaction between the electron lone pair of sulphur atoms with metal. Other less-known applications of 2TC have also been reported. For example, a series of acyclic and macrocyclic nucleobase derivatives containing 2TC have been shown to be highly selective of the extraction of Ag^+^ ions [20].

The biological functions of 2TC depend on its interaction in a water environment. Thus, interactions of explicit water molecules with the thiocarbonyl group of 2TC have been reported to enable water–chromophore electron transfer (WCET), which leads to intersystem crossing formation [21]. Microsolvation effects on the excited state deactivation dynamics of 2TC have also been studied using FTIR, Raman, and UV-Vis spectroscopies [22]. Clustering in water and methanol may prohibit or promote an excited state proton transfer reaction from the sulphur atom to the neighbouring nitrogen atom due to the hydrogen bonding chain between 2TC and protic solvent molecules [23]. However, how hydration affects the tautomerism of 2TC is also of interest, and for this reason, it has been the subject of several ab initio studies [21,24,25], but water-assisted tautomerism has only been studied in the cytosine molecule [26,27]. In the present study, the effect of the first and second hydration shells with 30 explicit water molecules on the molecular geometry of 2TC and its tautomerism were analysed and compared with cytosine at the same level. To determine how hydration modifies the geometric parameters and charges, it appears to be of interest to know the reactivity of this molecule in its bonding with DNA.

Vibrational studies on 2TC in inert Ar and nitrogen gas matrices [28,29], in KBr pellets [30], and in polycrystalline films have been reported [28,29]. In these studies, the band assignments have not been completely and accurately analysed in detailed form as in the present manuscript. Owing to the biological importance of 2TC, it is worthwhile to perform a new detailed investigation of its vibrational spectra and its comparison with that of cytosine molecules. For this task, specific and better scaling procedures for the wavenumber calculations were carried out, together with solid-state simulations of the crystal unit cell through several tetramer forms of 2TC. In the spectra of solid-state samples, several bands are considerably shifted due to intermolecular hydrogen bonds (H-bonds) and therefore various bands were not assigned accurately in the previous work [30]. An attempt has also been made here to improve their assignment by optimising several tetramer forms of 2TC so that the obtained spectra can be better compared with the experimental ones in the solid state. Thus, a remarkable improvement in the results reported previously was achieved.

The *keto* tautomer T1 of cytosine (C) is the main form present in the DNA helix. A specific guanine: cytosine (G:C) Watson–Crick (WC) pair formation appears to be responsible for genetic code maintenance. When C is substituted by another nucleobase, this leads to a wrong genetic code introduction. Therefore, how 2TC affects these canonical WC and reverse pairs (rWC) with G in its structure and stability will be determined in the present study. The stability of the 2TC:G pair is expected to be lower than the one related to the canonical ones, C:G, which suggests that replacing cytosine with 2TC would worsen the speed and fidelity of non-enzymatic copying in the formation of RNA templates. In this study, this effect will be confirmed in a few optimised DNA–DNA microhelices with three nucleoside base pairs.

2TC presents unique abilities to modify DNA, which may be relevant in the design of DNA-binding drugs with high antitumor efficacies. For this purpose, all information about its molecular properties, reactivity, and tautomerism appears of interest to explain its important pharmaceutical applications. It can act as an anticancer agent [25,31] or through its derivatives [32] or nucleoside form 2-thiocytidine [33]. The cytotoxic activities of some forms were found to be even higher than that of cisplatin, and they were even active against cisplatin-resistant cell lines [34]. Sulphur metabolic pathways are essential for survival and expression of virulence in many pathogenic bacteria, including Mycobacterium tuberculosis [35], and thus, 2-TC may represent a valuable lead for antibacterial and antituberculosis drug development.

The aims of the present study were: (i) To determine the effect of sulphur instead of oxygen on the molecular structure of 2TC in an isolated state, solid state and water solution; (ii) to analyze the effect of the water solvent on the geometrical parameters, molecular properties, and tautomeric equilibrium; (iii) to characterize the normal modes of 2TC and to accurately assign the experimental vibrational spectra of 2TC in the gas phase through several tautomer forms in the isolated state and in the solid state sample through several tetramer forms; (iv) to compare the effect of a sulphur atom instead of oxygen on the WC pair with guanine; and (v) to describe the effect of 2TC on DNA–DNA optimised microhelices through their calculated helical parameters, as compared to the related canonical ones.

## 2. Results and Discussion

### 2.1. Isolated State

#### 2.1.1. Relative Stabilities of 2TC Tautomers

Cytosine tautomerization has been widely analyzed both experimentally [36,37,38] and theoretically [39,40,41,42]. Theoretical studies of isolated cytosine have revealed a diversity of possible tautomers, six of which have relatively low energy, with three in the low range of <7 kJ·mol^−1^, and another three in the 10–40 kJ·mol^−1^ range of higher energy. The relative stabilities of these tautomers depend on the method used and basis set, and they also show significant dependence on the environment. Thus, studies indicate that in the isolated state, they appear as a mix of *amino-keto* and *amino-hydroxyl* tautomers. In DNA/RNA helices, the *amino-keto* tautomer (**T1**) (also identified as the *amino-oxo* form) is the main form of cytosine, the “canonical” one.

**Figure 1:**
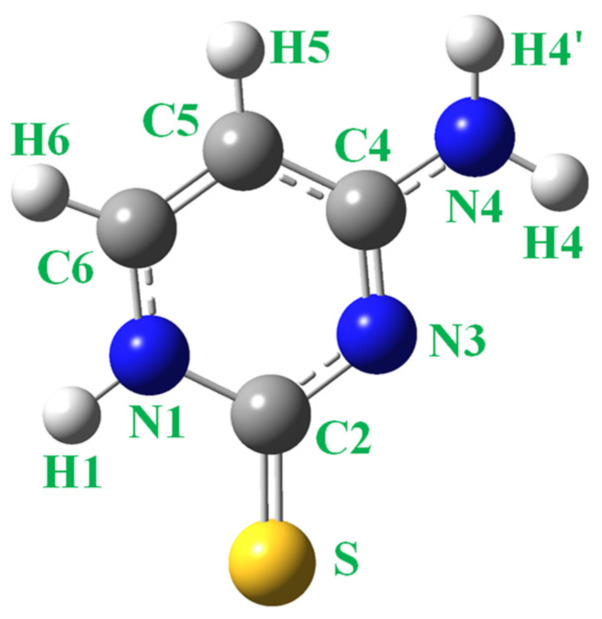
Labelling of the atoms in the 2-thiocytosine molecule.

**Figure 2:**
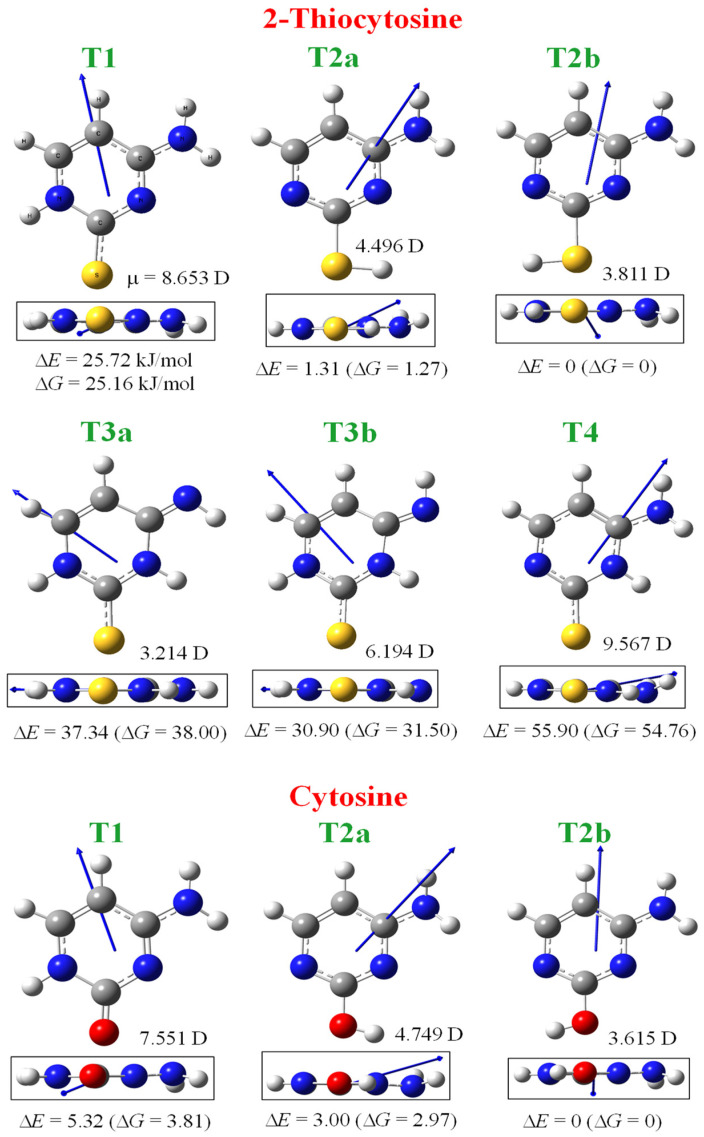
Optimised structures of 2-thiocytosine and labelling of tautomers: (T1) 1H amino-thione form, (T2a) amino-thiol *trans* form, (T2b) amino-thiol *cis* form, (T3a) imino-thione *cis* form, (T3b) imino-thione *trans* form, and (T4) 3H- amino-thione. In the three most stable tautomers of the cytosine molecule, the sequence is as follows: (T1) oxo form, (T2a) hydroxy *trans* form, and (T2b) hydroxy *cis* form. The dipole moment (μ) and its vector in blue color, as well as the increment of energy + ZPE (ΔE) and Gibbs energy (ΔG), are also included. Nitrogen atoms are shown in blue and oxygen atoms in red color.

Unlike the wide analysis of cytosine tautomerism, 2TC has been much less studied. Experimentally, several amino-thiol tautomer forms have been detected in its IR spectrum in inert gas matrices [28,29], while only the T1 tautomer appears in KBr pellets [30] and in polycrystalline films [28,29]. Theoretical studies have been reported at the semiempirical [43] and ab initio levels [25,28,29], although at a lower level than the one in the present manuscript. The labelling of atoms is indicated in Figure 1 according to the standard notation used for cytosine and its derivatives [1,2], while the plot with their optimised structures is shown in Figure 2. For comparison purposes, the plots of the three most stable optimised tautomers of the cytosine molecule determined at the same MP2/6-31G(d,p) theoretical level are also included in this figure, as well as the difference in total energy (ΔE), with zero-point energy correction (ZPE) and the Gibbs free energy (ΔG).

Table 1 shows a comparison of the gas phase relative energies in 2TC vs. those of cytosine. It is clear from this table that the **T2b** tautomer is the most stable in both molecules. The entropy effect on the Gibbs free energy has been observed to be very small and practically of no significance for its tautomeric equilibria, with the enthalpic form being dominant in the equilibrium constant. Cytosine is the only canonical nucleobase with the **T2b** *enol* form more stable than **T1** *keto* in the isolated state. Thus, great interest arises in the tautomerism of cytosine and its derivatives, which is analysed herein in several media.

The sulphur atom in 2TC did not show any effect on the stability trend of cytosine tautomers, with it being the same in both 2TC and cytosine molecules: **T2b** > **T2a** > **T1** > **T3b** > **T3a** > **T4.** Because **T2a** is almost as stable as the amino 2-hydroxy *cis* form (**T2b**), both tautomers can appear in the gas phase. However, these **T2a** and **T2b** tautomers have little biological relevance because they involve the deprotonation at the N1 position where the furanose ring is attached through the biochemical machinery of the cell to build the nucleoside form.

Tautomer **T1** in 2TC is almost four times less stable than the related one in cytosine, due to the noticeably weaker C-S bond than C=O, indicating that this tautomer will not be present in the gas phase sample of 2TC. In the amino tautomers of cytosine, the NH_2_ group is more strongly bonded to the heterocyclic ring, 1.369 Å at the MP2/6-31G(d,p) level (1.371 Å with MP2/6-31+G(d,p)) in tautomer **T1**, than to the benzene ring in the aniline molecule, 1.406 Å at the same MP2 level [46]. Thus, a shorter C4-N4 bond in cytosine leads to lower pyramidalization of the NH_2_ group, with lower inversion (ω) and tilt angle (ε) values than in the aniline molecule, ω = 47.18° and ε = 4.37°, and this also leads to slightly shorter N4-H-bonds, 1.008 Å in cytosine compared to 1.010 Å in aniline at the same MP2 level [46].

This tautomer of 2TC has a higher HOMO energy orbital value and lower LUMO energy value than those of cytosine. The narrower HOMO–LUMO gap in 2TC than in cytosine shows that 2TC is more polarizable with higher chemical reactivity than cytosine. Thus, cytosine, which is harder (with a larger chemical hardness η), should be less reactive than 2TC in unimolecular reactions such as isomerization, dissociation, and radical formation. This information could be helpful when attempting to rationally explain the activity of 2TC in biological and chemical reactions. The enhanced reactivity of 2TC compared to cytosine suggests the probable occurrence of point mutations in which cytosine is replaced by 2TC, leading to reduced stability of DNA [31].

**T3a** and **T3b** imino tautomers are always less stable than the corresponding amino forms, and they appear noticeably more planar than the amino ones due to their NH_2_ pyramidalization. The imino group is full planar with the cytosine ring, with a N3=C4-N-H4 torsional angle of 0.0° and C2-N3=C4-N of 180.0°, which is due to a remarkable shortening of the C4-N4 bond length (1.292 Å by MP2) as compared to the amino tautomers. This shortening of the C4-N4 bond leads to a less flexible imino group to form WC pairs with complementary guanine. Moreover, the positive charge on H4 (0.370*e*) and H4′ (0.379*e*) imino hydrogens is lower than that on the amino tautomers (0.449*e* in H4 of T1), and the negative charge on N4 is considerably lower in the imino tautomers (−0.777*e* in T3a/−0.789*e* in T3b) than in the amino ones (−0.899*e* in H4 of T1). Therefore, little reactivity is expected for these tautomers if they are occasionally formed in the DNA or RNA helices.

The tautomeric equilibrium and, therefore, the stability trend of tautomers strongly depend on the chemical environment, differing in the solid state [47] and in the solution phase [2]. This tautomeric equilibrium also depends on how the compound is absorbed on the metal surface. Therefore, tautomer **T1** of 2TC was calculated to be the most stable when it is adsorbed on gold nanoparticle surfaces [17]. If the tautomers are selectively excited, they can be converted into another by proton transfer or through proton rotation of 180° degrees. Thus, in 2TC, the conversion of one amino-thiol tautomer into another by rotation of 180° degrees of the SH group was observed through neutron diffraction [38].

#### 2.1.2. Dipole Moments

The computed dipole moments (μ) of the main six tautomers of 2TC and the three most stable cytosines are also shown in Figure 2, together with the dipole moment vector. The calculated value of μ in the **T1** tautomer of cytosine, 7.551 D, appears to be in good accordance with the experimental one, 7.0 D [48]. Although experimental gas phase values of μ are not available for 2TC, the good accordance obtained with cytosine permits us to suppose that our calculated μ values will be close to the expected experimental ones.

**T1** and **T4** tautomers in 2TC appear with the highest μ values and therefore, it is expected that they are the main forms in hydrated environments. This feature may facilitate its presence and interaction in many biological processes, including that with DNA. Although the μ value is noticeably higher in tautomer **T1** of 2TC than in the cytosine molecule, H-bonds through the sulphur atom with the surrounding water molecules are expected to be weaker in 2TC than through the oxygen atom of cytosine.

Also, the **T2b** tautomer, as well as **T3a**, **T3b,** and **T4**, has a higher value of μ in 2TC than in cytosine, but with values noticeably lower than in **T1**, and therefore, they are less likely to be present in water solution. The sulphur atom’s position on 2TC does not seem to significantly change the direction of the dipole moment vectors plotted in Figure 2, as compared to the cytosine molecule. This direction appears out-of-plane in the **T1**, **T2a**, **T2b**, and **T4** tautomers of 2TC, especially in **T2b**, which is perpendicular to the ring plane.

#### 2.1.3. NBO Charges

The negative charge on the sulphur atom is remarkably lower than on oxygen, and this negativity only appears in **T1** and **T4** thione forms. However, it changes to a very small positive value in thiol forms. Because of its lower electronegativity than oxygen, sulphur substitution gives rise to a large decrease in the positive C2 atomic charge where the sulphur atom is bonded. This C2 charge slightly increases in **T2a** and **T2b** thiols and **T3a** and **T3b** imino tautomers compared to **T1** and **T4** thione forms. As a consequence of this sulphur substitution at C2, the negative charges of the neighbour N1 and N3 nitrogens experience a large decrease because of the lower electronic charge flow from C2 to these atoms.

Compared to cytosine, the presence of a sulphur atom instead of an oxygen substituent at the C2 position is crucial because the presence of this sulphur allows 2TC to form much weaker hydrogen bonds that are reversible, which, in turn, facilitates biological processes such as enzyme–substrate interactions, and can be used for regulation in metabolic processes [31]. In contrast, sulphur is better than oxygen at stabilizing the structures in the solid state through electron donation, as explained below.

### 2.2. Molecular Structure in the Solid State of 2TC

The crystal unit cell found using X-ray analysis is composed of two independent molecules in only the **T1** tautomeric form and linked in pairs by two strong N1-H···N3 and N4-H···S hydrogen bonds in a very nearly planar arrangement [47]. These two molecules have almost the same structure in the crystal, with the largest difference of only 0.002 Å being in the bond lengths and 1.5° in the N1-C2=S angle (Table 2). Both molecules are also nearly planar, with small deviations of planarity from the NH_2_ group.

**Figure 3:**
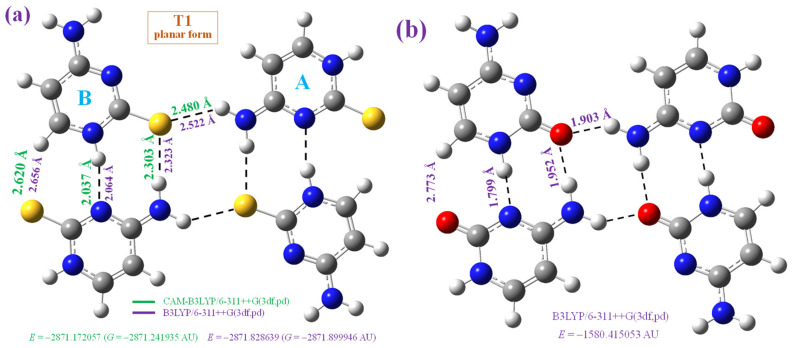
Optimised planar tetramer forms at the B3LYP/6-311++G(3df,pd) and CAM-B3LYP/6-311++G(3df,pd) levels with (**a**) 2TC molecule and (**b**) cytosine molecule. The notation in light blue c in 2TC corresponds to that in the X-ray study [47] related to molecules A and B.

**Figure 4:**
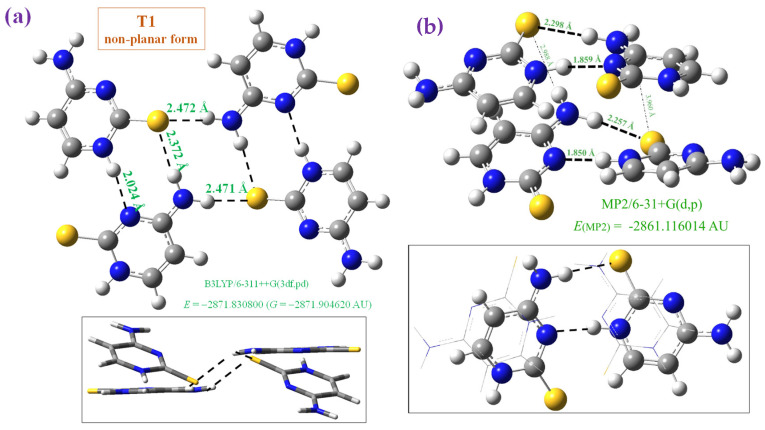
Optimised non-planar tetramer forms with 2TC: (**a**) at the B3LYP/6-311++G(3df,pd) level and (**b**) at the MP2/6-31+G(d,p) level (stacking form).

We optimised this crystal unit cell as reported by X-ray data [47] through a tetramer form at four theoretical levels: B3LYP/6-31G(d,p), B3LYP/6-311++G(3df,pd) in Figure 3a and Figure 4a, CAM-B3LYP/6-311++G(3df,pd) in Figure 3a, and MP2/6-31+G(d,p) in Figure 4b. However, the planar arrangement reported for the crystal is a saddle point in the potential energy surface in the calculation using both B3LYP and CAM-B3LYP methods (Figure 3a). In contrast, the non-planar form optimised at the B3LYP/6-311++G(3df,pd) level and plotted in Figure 4a corresponds to the true minimum, as well as the optimised structure at the B3LYP/6-31G(d,p) level. In this last case, the deformation is very large. We tried to optimize the planar form at the MP2/6-31+G(d,p) level, but it was not stable and the final structure obtained and included in Figure 4b shows an almost stacking form between the dimers of the tetramer system. The main geometrical parameters optimised in these tetramer forms are presented in Table 2, in which the values of molecule A and molecule B (in parenthesis) are included and compared to the experimental one, in the last column. These deformed optimised structures obtained in 2TC are in contrast to the full planar form optimised in cytosine (Figure 3b). The calculated CP-corrected interaction energies in several of these tetramer forms are shown in Table 3. An analysis of these optimised tetramer forms is as follows:

Planar form, Figure 3a: Its planarity is in agreement with that reported for the crystal, and both B3LYP and CAM-B3LYP methods give rise to this planarity. Moreover, their calculated geometric parameters are, in general, more in accordance with the experimental data than those found in the non-planar form. The computed intermolecular H-bond lengths between the four tetramer molecules appear shorter when using the CAM-B3LYP method than when using B3LYP, and with both methods, they are noticeably shorter than those found in the crystal. Therefore, N4-H···S is 2.323Å with B3LYP vs. 3.345 Å in the crystal and N1-H···N3 is 2.064Å vs. 3.022Å in the crystal. This large difference in the H-bonds is also observed in the other optimised tetramer forms. The intermolecular contact C6-H···S between molecules A and B is also shorter by B3LYP, 2.656 Å vs. 2.69 Å in the crystal, which also appears shorter than the normal van der Waals distance between sulphur and hydrogen atoms, 2.9 Å. The deformation energies were found to be lower in the tetramer forms with 2TC than with cytosine, as expected, and therefore, the E^int^ and ΔE^CP^ energies were also lower than with cytosine.

The NH_2_ group is coplanar with the cytosine ring when it is intermolecularly H-bonded to the sulphur atom, but it has small pyramidalization when it is not H-bonded. This is due to the H-bond shortening the C4-N4 bond, increasing the sp^2^ character of the NH_2_ group, i.e., increasing its planarity. In general, the optimised values in the planar tetramer forms agree well with the experimental ones, especially in the C-N bond lengths, with the values with B3LYP being slightly better than with CAM-B3LYP.

Non-planar form, Figure 4a: This tetramer is a true minimum with the four molecules in different planes but with the same H-bonds as that in the planar form. The H-bond N4-H···S is now weaker, 2.372 Å vs. 2.323 Å in the planar form, while N1-H···N3, it is slightly stronger, 2.024 Å vs. 2.064 Å in the planar form. This difference only slightly affects the optimised geometric parameters, which appear close to that in the planar form, Table 2, and also in good accordance with the experimental values. NH_2_ appears with slight pyramidalization due to the small lengthening of the C4-N4 bond. Using a lower basis set, such as at the B3LYP/6-31G(d,p) level, the deformation is remarkably higher and far from that found in the crystal. The deformation and interaction energies are slightly higher than the corresponding planar form.

Stacking form, Figure 4b: This tetramer form was only obtained with MP2. The planar and non-planar forms were not stable during optimization with this method. This system appears to be stabilised by the same intermolecular H-bond types as in other tetramers, but in two planes in stacking form, with a slightly weaker N4-H···S H-bond and noticeably stronger N1-H···N3 bond. In addition, π-π interactions stabilize this system more. Due to this arrangement, the C4-N4 bond is longer, leading to a noticeable increment in the NH_2_ group pyramidalization. The optimised geometrical values also appear to be in agreement with the experimental results, especially in the C-N bonds, but less than with B3LYP and CAM-B3LYP. This stacking pattern has also been found in other thiopyrimidines [49].

Significant differences can be observed when comparing 2TC and cytosine tetramer forms. The geometrical arrangement is basically the same, but the remarkably lower electronegativity of the sulphur atom than the oxygen gives rise to intermolecular hydrogen bonds weaker with 2TC than with cytosine and coplanarity loss between molecules. As expected, the C-S area is the most affected, with an experimental C-S bond of 1.701Å [47], which differs from the C=O double-bond character of 1.24 Å in cytosine. This gives rise to a shortening of neighbour N1-C2 and C2-N3 ring bonds. These changes induced by sulphur substitution have been explained [47] as the result of bonding electrons flowing from the outer double bond to the neighbouring C2-N3 bond and especially N1-C2. An analysis of the NBO atomic charges indicates that this flux is mainly from the C5 and N4 atoms.

**Figure 5:**
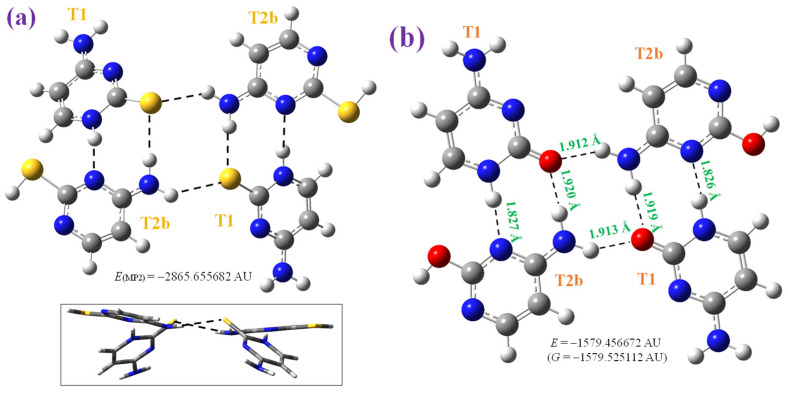
Optimised tetramer form with a mixture of the T1 and T2b tautomers in (**a**) 2TC at the MP2/6-31G(d,p) level and (**b**) cytosine at the B3LYP/6-31G(d,p) level.

Because the X-ray technique cannot determine the hydrogen atom positions well, another aim of the present study is to analyse the possible fact that a mixture with **T1** and **T2b** tautomeric forms could exist in the 2TC crystal. For this purpose, a tetrameric form with this mixture was optimised at the MP2/6-31G(d,p) level and are plotted in Figure 5a. The H-bond arrangement appears to be the same as with the **T1** tautomer and with a long non-planarity, but the stacking form was not achieved in this case. This mixed tetramer form was also optimised at the M06-2X/6-311++G(3df,pd) and B3LYP/6-31G(d,p) levels. The H-bond arrangement is maintained with both DFT methods. However, the crystal packing forces find it difficult to maintain this non-planarity, thus the existence of these mixed tetrameric forms in the solid state.

These non-planar optimised structures with 2TC differ from the full planar form obtained at the B3LYP/6-31G(d,p) level with the cytosine molecule (Figure 5b). Therefore, these tetramer forms with cytosine appear to be available to be formed in the solid state, although this was not observed in the IR and Raman spectra analyses. With the help of all of these optimised forms, the bands found in the recorded experimental IR and Raman spectra in the isolated and solid state can be accurately assigned.

### 2.3. Vibrational Analysis in the Isolated State of 2TC

**Figure 6:**
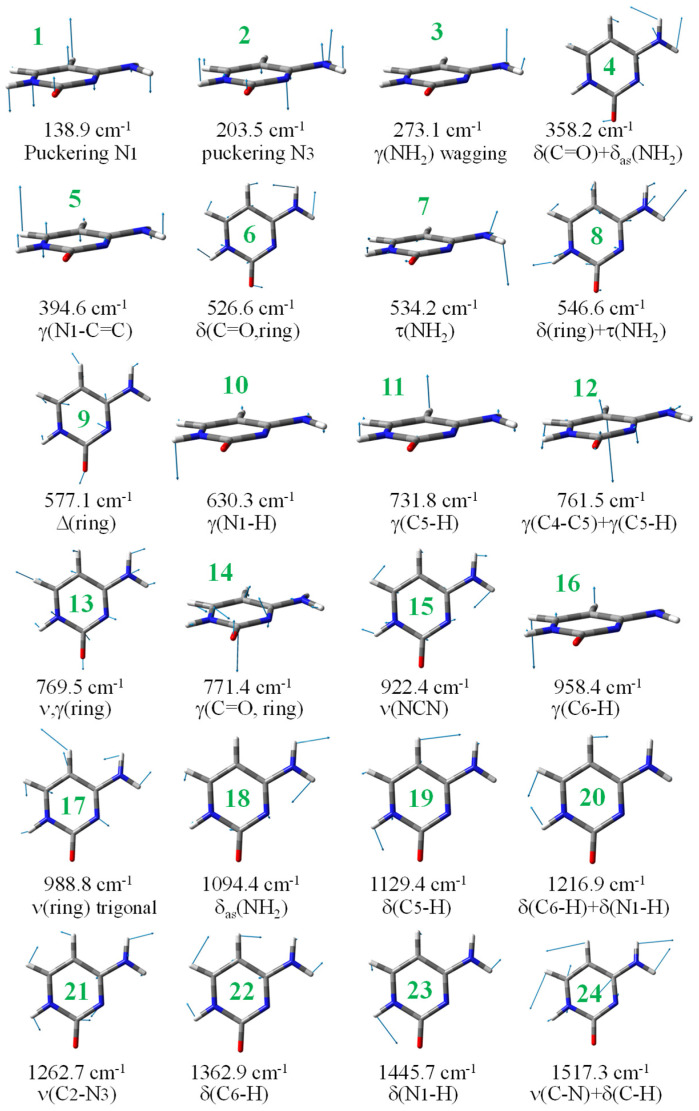
Characterization of the normal ring modes in tautomer T1 of the cytosine molecule at the B3LYP/6-31+G(d,p) level, together with the calculated wavenumbers and the main assignment.

The first task for an accurate assignment of the experimentally observed bands in 2TC was to identify the normal ring modes in the cytosine molecule, which are plotted in Figure 6. Therefore, through comparison of the displacement vectors plotted in this figure with those obtained in the calculated wavenumbers of 2TC, an accurate assignment of each vibration and a direct relationship with the cytosine molecule can be made. In addition, this will permit trends to be established with related cytosine derivatives.

This kind of characterization shown in Figure 6 was performed a long time ago by Varsany [50] following the Wilson notation [51] for the benzene ring modes [52] and for uracil molecules [53].

**Figure 7:**
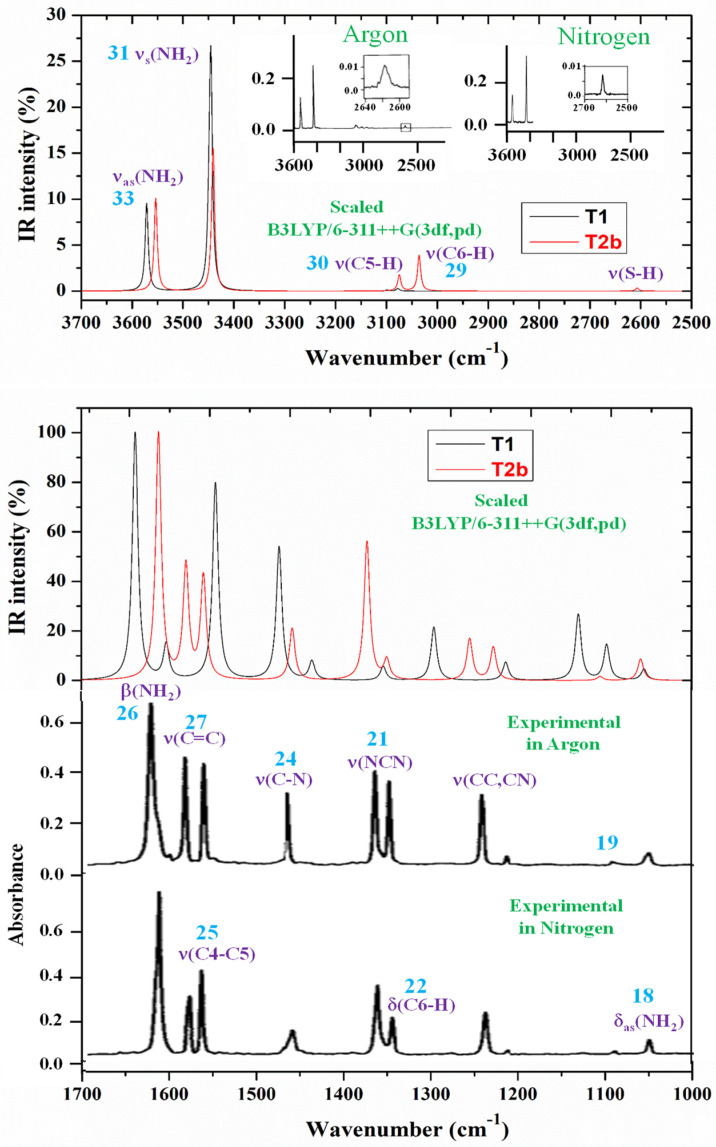

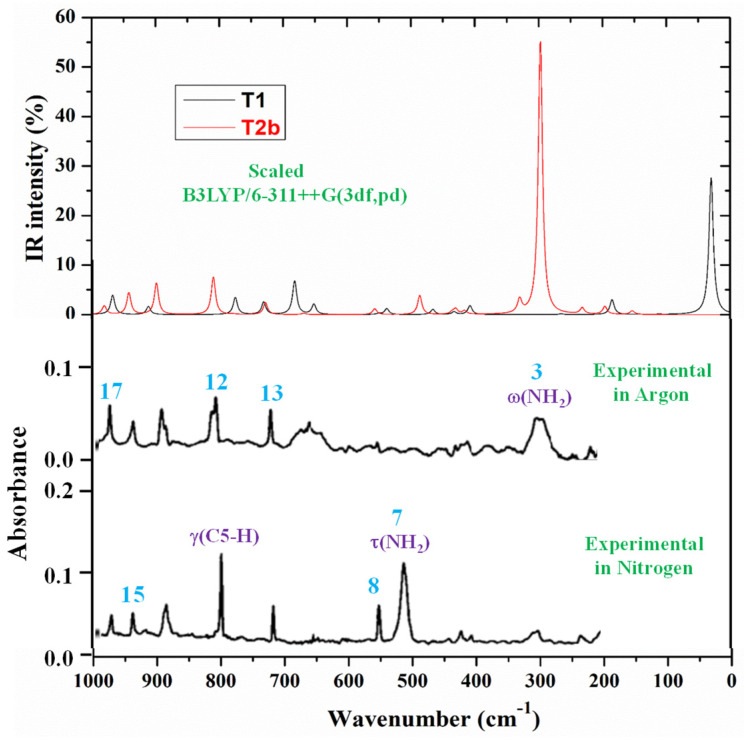
Theoretical scaled IR spectra in the monomer form of T1 and T2b tautomers of 2TC in the 3700–2500 cm^−1^, 1700–1000 cm^−1^, and 1000–0 cm^−1^ ranges, together with the experimental ones in low-temperature inert gas matrices: Ar matrix isolation [28,29] and nitrogen matrix [28]. The main assignments (in violet color) of the normal modes correspond to tautomer T2b, while the blue color indicates the number mode characterized in Figure 6.

The theoretically scaled IR spectra of **T1** and **T2b** tautomers of 2TC with the corresponding experimental ones in Ar matrix isolation [28,29] and in the N_2_ matrix are included in Figure 7 in the 3700–2500, 1700–1000, and 1000–0 cm^−1^ ranges. This spectral comparison can facilitate the band assignments. In this figure, the numbers in blue colors on the experimental bands correspond to our assignment according to the displacement vectors of the ring modes described in Figure 6 for the cytosine molecule. The main assignment in violet color is also included in the strongest experimental bands.

Wavenumbers computed using theoretical DFT methods are overestimated for many reasons [52,54]. To rectify this overestimation, several specific scaling procedures have appeared in the bibliography for each method and basis set [52,53,54,55,56], achieving a notable improvement in the wavenumber values. Nowadays, it has become a common procedure used by many authors to achieve accurate assignment of experimental bands. Among these available procedures, the Linear Scaling Equation (LSE) and the Polynomic Scaling Equation (PSE) seem to be the most appropriate, with a slight improvement with PSE. In the present study, the polynomic scaling equation procedure (PSE) was mainly used to scale the calculated wavenumbers.

ν^scal^ = −6.8 + 1.0076·ν^cal^ − 1.38 × 10^−5^ (ν^cal^)^2^  by B3LYP/6-311++G(3df,pd)

ν^scal^ = −10.6 + 0.9904·ν^cal^ − 0.955 × 10^−5^ (ν^cal^)^2^ by CAM-B3LYP/6-311++G(3df,pd)

The scaled frequencies with their relative (%) IR intensities in the **T1** tautomer (first two columns) are presented in Table 4, while the calculated and scaled values and their relative IR intensities in **T2b** are listed in the third to fifth columns. The experimental frequencies with the absolute IR intensities in Ar and N_2_ matrices [28,29] and the main assignments reported by Rostkowska et al. in these refs. [28,29] are also included. The notation utilised by these authors for the normal modes has been changed to allow for better comparison with the new and more standard notation applied in the present work. Some changes have also been made to the experimental wavenumber assignments to update them with the new notation. The last two columns show our assignments, including the numbers of the normal modes characterised in Figure 6, and the potential energy distribution (PED, in %) in parenthesis. Contributions lower than 10% were not included. Because the scaled values in **T2a** appear almost the same as in **T2b**, with differences in general lower than 1 cm^−1^, the results with **T2a** are not included in Table 4.

Comparing the scaled wavenumbers with the experimental ones, it was noted that almost all scaled values in **T2b** (marked in red in Table 4) agree well with the experimental bands in both frequency and intensity. In contrast, the scaled values in the **T1** tautomer differ markedly from the experimental ones. Only a few wavenumbers, marked in red in Table 4, agree well. Moreover, the highest IR intensity calculated in the vibration at 1639 cm^−1^ in the **T1** tautomer differs in ca. 20 cm^−1^ compared to the experimental ones at 1611 (Ar)/1619 cm^−1^ (N_2_), while the scaled wavenumber in **T2b** at 1612 cm^−1^ agrees perfectly with the experimental ones. In addition, the second strongest IR intensity vibration, scaled at 1546 cm^−1^ differs in ca 15 cm^−1^ compared to the experimental ones at 1562 (Ar)/1558 cm^−1^ (N_2_), while the scaled value in **T2b** at 1561 cm^−1^ agrees perfectly with the experimental values. There are also predicted vibrations in the **T1** tautomer that do not have a relationship with the experimental values, and vice-versa, with experimental bands that are not predicted in this **T1** tautomer. The appearance of these features with most of the scaled values in **T1** allows us to assert that in Ar and nitrogen matrices, only **T2b** (and **T2a**) tautomers exist. The **T2b** tautomer is the only one present in the gas phase, and therefore, the assignments included in the last two columns correspond only to this **T2b** tautomer, in agreement with ref. [28]. This conclusion in 2TC is also in agreement with a lower stability of **T1** (24.03kJ/mol) than **T2b**, while the difference is ca. 6kJ/mol in cytosine. Therefore, the scaled wavenumbers of the **T1** tautomer were not used for the assignment of the experimental bands. Most of the assignments shown in Table 4 are in agreement with those reported in the 2TC molecule [28,29].

Although the spectra shown in Figure 7 and characterized in Table 4 are self-explanatory, our scaled wavenumbers in tautomer **T2b** are in excellent agreement with the experimental values. A brief analysis of several normal modes is as follows:

For thio-derivatives of nucleic acid bases, the C-S stretching mode is among the most important vibrations as this mode indicates the thiol population versus thione form. However, this mode has not been clearly identified in the theoretical calculations of 2TC.

The S-H vibrations can be clearly identified in tautomer **T2b**. Therefore, its ν(S-H) stretching mode occurs as pure mode with 100% PED in the scaled wavenumber at 2608 cm^−1^ and in good agreement with the very weak IR bands at 2617 cm^−1^ in Ar and at 2615 cm^−1^ in the N_2_ matrix. The in-plane bending mode were identified in the scaled values at 942 cm^−1^ and 899 cm^−1^ and is correlated excellently with the experimental IR bands in the 888–946 cm^−1^ region. The out-of-plane γ(S-H) vibration is clearly identified as pure mode at 329 cm^−1^, but with very low IR intensity, and therefore, it was not related to any experimental band.

The atomic displacement vectors of the scaled wavenumber at 1255 cm^−1^ were not identified with any cytosine normal mode, as shown in Figure 6. However, they clearly correspond to a Kekule vibration, as mode 14 in the benzene molecule [50,52], and as such, it was assigned in Table 4. The scaled wavenumber at 980 cm^−1^ was identified as mode 17 of the cytosine molecule, although its displacement vectors clearly correspond to trigonal vibrational, such as mode 12 in the benzene molecule.

### 2.4. Vibrational Analysis in the Solid State of 2TC

**Figure 8:**
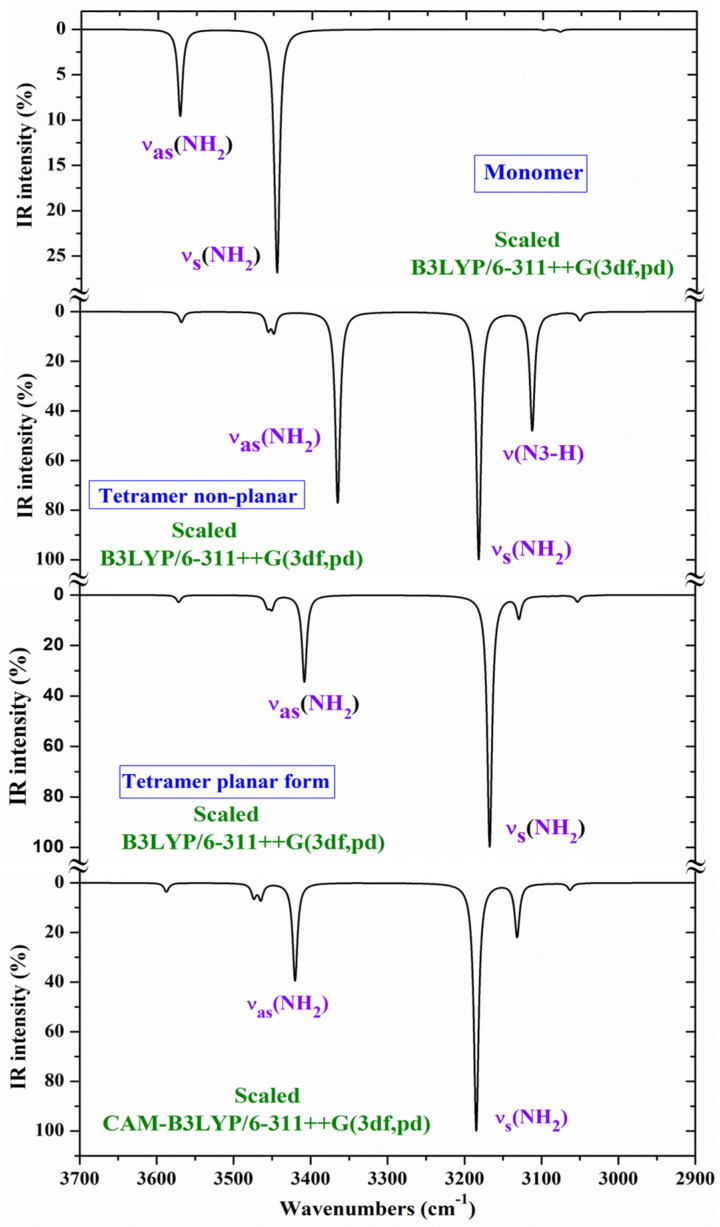
Theoretical scaled IR spectra in the 3700–2900 cm^−1^ range of three optimised tetramer forms of tautomer T1 of 2TC using the PSE procedure, together with that determined in the isolated state (monomer form). The main assignments are shown in violet color.

**Figure 9:**
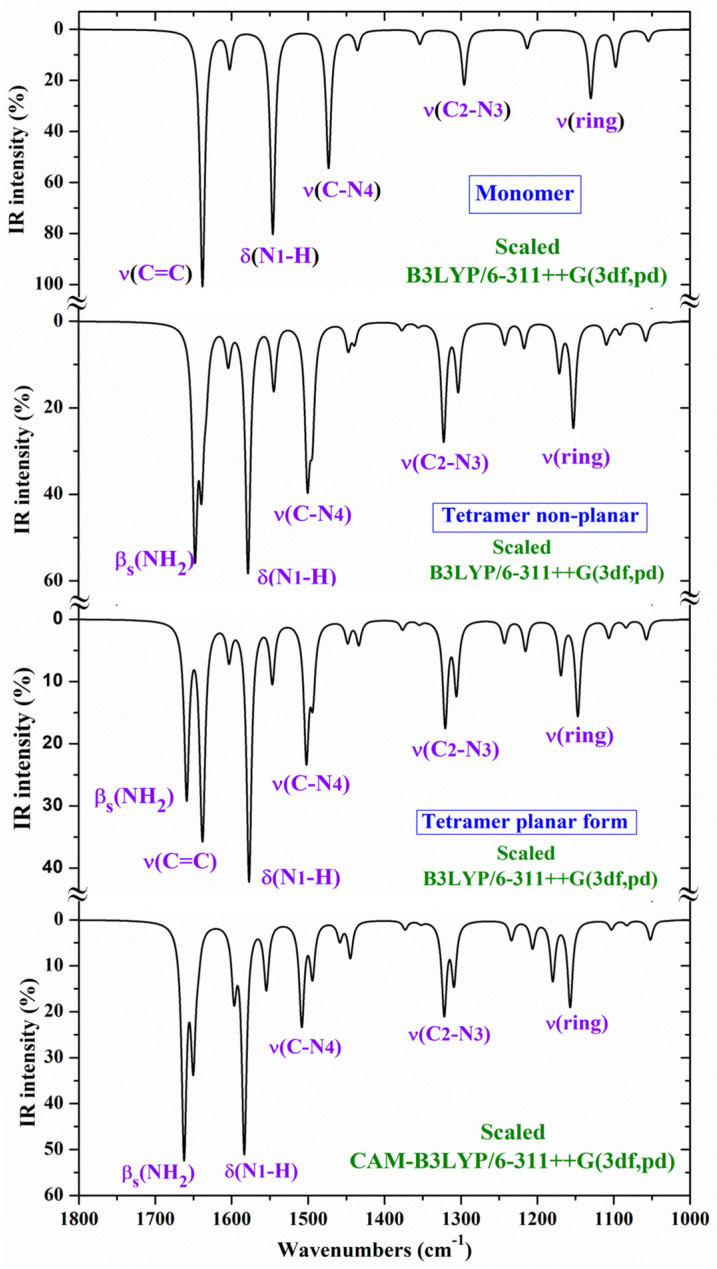
Theoretical scaled IR spectra in the 1800–1000 cm^−1^ range of the three optimised tetramer forms of tautomer T1 of 2TC using the PSE procedure, together with that determined in the isolated state.

**Figure 10:**
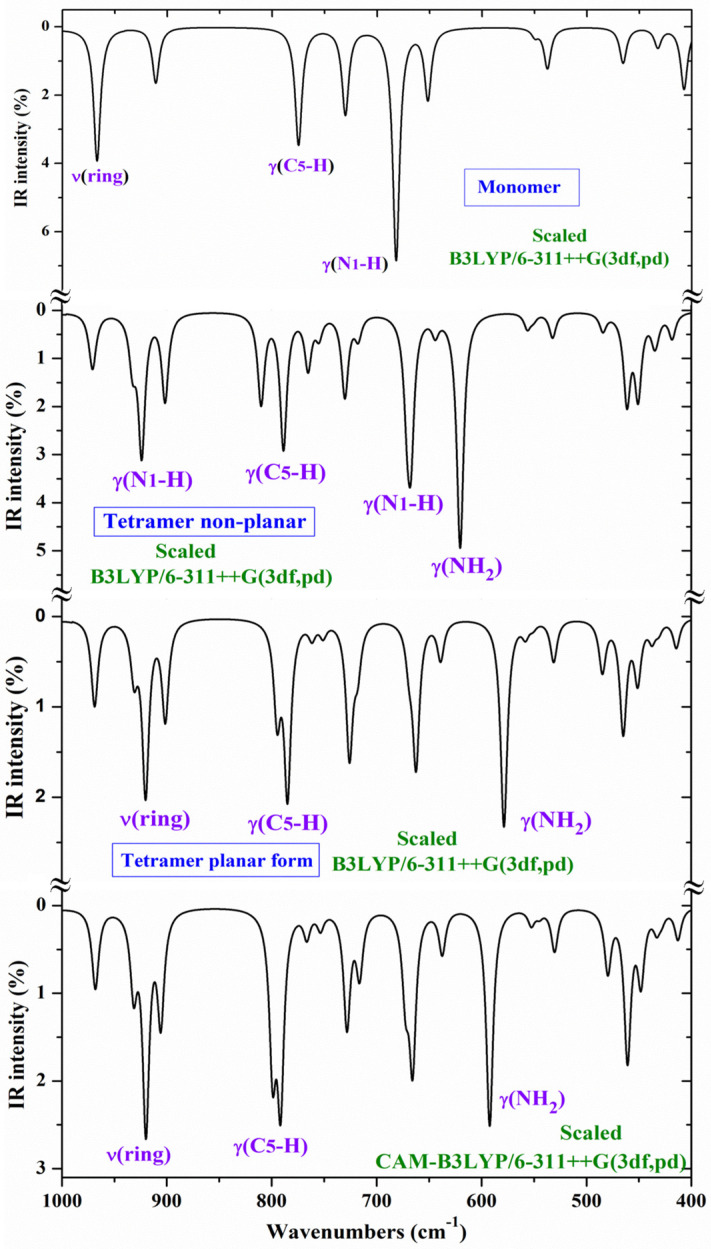
Theoretical scaled IR spectra in the 1000–400 cm^−1^ range of the three optimised tetramer forms of tautomer T1 of 2TC using the PSE procedure, together with that determined in the isolated state.

The experimental IR spectrum has been recorded in thin polycrystalline films [28], as well as in KBr pellets [30]. In these spectra, only the **T1** tautomer has been reported to be detected. For an accurate and better assignment of these spectra, several tetramer forms with **T1** have been previously optimised (Figure 3a and Figure 4a). The polynomic scaling equation procedure (PSE) was only used to scale the calculated wavenumbers. To facilitate the analysis and assignment of the experimental bands, the theoretical scaled IR spectra were divided into three ranges, 3700–2900 cm^−1^ (Figure 8), 1800–1000 cm^−1^ (Figure 9), and 1000–400 cm^−1^ (Figure 10). For comparison purposes, of the two experimental IR spectra available, the spectra recorded in polycrystalline films [28] appeared to have better quality and therefore, they were mainly discussed in the present study.

The FT-Raman spectrum was experimentally recorded in the 3700–0 cm^−1^ range. For an accurate assignment of bands, the scaled Raman spectra of three tetramer forms of the T1 tautomer were plotted and analysed in detail. The PSE procedure was also used for scaling. For simplicity, these spectra were not included in the present manuscript

Comparing the scaled IR and Raman spectra in the three tetramer forms, it is noted that those obtained with the B3LYP and CAM-B3LYP methods in the tetramer planar forms appear the same. Thus, for simplicity, the scaled values at the CAM-B3LYP/6–311++G(3df,pd) level (planar form, Figure 3a) were only included in Table 5 with their relative IR and Raman intensities (first three columns), while the scaled values in the non-planar tetramer form obtained at the B3LYP/6-311++G(3df,pd) level are listed in the fourth to sixth columns (Figure 4a). Four scaled wavenumbers appear for each vibration, corresponding to the four tetramer molecules. Because of the symmetry of the system, the wavenumbers of these four molecules will be very close, and to avoid repetition, differences in the scaled values lower than 1 cm^−1^ were not considered and they were not included in Table 5.

Of the four wavenumbers for each vibration, a special identification was made because two of them may have modes involved in intermolecular H-bonds, and the other two may not. This is due to the amino group and the sulphur atom appearing in the external position of the optimised tetramer system. These wavenumbers of interest were included in Table 5, and those with the highest IR intensity are included in bold type, and those with the highest Raman intensity are shown in *italic* type. These wavenumbers were only considered and their IR/Raman intensities were included in the Table. The next two columns correspond to the experimental wavenumbers in the IR and Raman spectra with their absolute IR and Raman intensities in parenthesis. Finally, the last two columns correspond to the main characterization of these vibrations, including the numbers of the normal modes plotted in Figure 6 and the %PED in parenthesis corresponding to the scaled values in bold type. Contributions lower than 10% were not included. Because of the close values between B3LYP (non-planar) and CAM-B3LYP (planar) and to avoid repetitions, the characterization included in the last two columns only corresponds to B3LYP. The main difference between both methods corresponds to only %PED.

Although Table 5 and the IR spectra shown in Figure 8, Figure 9 and Figure 10 are self-explanatory, a brief analysis of several normal modes is included as follows.

**NH_2_ modes**: The antisymmetric stretching mode (ν_33_) appears as pure mode (100% PED), and in H-bonded groups, it was scaled with B3LYP at 3365 cm^−1^ (3420 cm^−1^ with CAM-B3LYP) for IR, in good accordance with the strong IR band at 3334 cm^−1^, and at 3353 cm^−1^ (3411 cm^−1^ with CAM-B3LYP) for Raman, also in good agreement with the shoulder observed in the experimental Raman spectrum at 3334 cm^−1^. The close experimental bands at 3312 cm^−1^ (IR) and at 3320 cm^−1^ (Raman) were also assigned to this mode but corresponded to NH_2_ groups with stronger H-bonds than in our tetramer system. The symmetric mode (ν_31_) was predicted at 3182 cm^−1^ with B3LYP, but no band was detected in the experimental spectra close to this value. Perhaps, it is involved in the strong band detected at around 3100 cm^−1^. This feature appears to be supported by the coupling of this mode (15% PED) with the N1-H stretching mode in the scaled wavenumber at 3112 cm^−1^ (IR) with B3LYP.

The scissoring β(NH_2_) mode (ν_26_) appears to be strongly coupled with the ν(C=C) mode (ν_27_). Due to the different %PED calculated for these modes with B3LYP and CAM-B3LYP, their assignments differ, appearing with the β(NH_2_) mode at a higher wavenumber than the ν(C=C) mode with B3LYP, and this assignment is reversed with CAM-B3LYP. Therefore, in this case, and due to the better accordance with the experimental values with CAM-B3LYP than with B3LYP, we changed the assignment of the last column. Thus, the scaled wavenumber at 1651 cm^−1^ with CAM-B3LYP was correlated well with the Raman line observed at 1669 cm^−1^, and the scaled value at 1644 cm^−1^ correlated with the IR band at 1645 cm^−1^

The torsional mode τ(NH_2_) (ν_7_) appears as almost pure mode (80% PED) in the predicted wavenumber at 532 cm^−1^ (with B3LYP) and at 530 cm^−1^ (with CAM-B3LYP), and it was correlated well with the experimental IR band at 527 cm^−1^. Another high contribution of this mode (66% PED) was predicted at 765 cm^−1^ (IR) with very weak IR and Raman intensity, and it was correlated well with the IR band at 752 cm^−1^ and the Raman line detected at 755 cm^−1^.

The wagging ω(NH_2_) (ν_3_) mode was also predicted as almost pure mode (84% PED) at 621 cm^−1^ (IR) with B3LYP and at 592 cm^−1^ with CAM-B3LYP and excellently correlated with the IR band at 594 cm^−1^. Its very weak Raman intensity predicted is in agreement with the very weak Raman line observed at 598 cm^−1^.

**C-NH**_2_ **modes:** The ν(C-N4) stretching appears identified as part of the ring modes 24 and 21 (Figure 6). The highest contribution is observed in mode 24 (ν_24_), which is scaled at 1501 cm^−1^ (IR) with B3LYP and at 1508 cm^−1^ with CAM-B3LYP, in good agreement with the strong IR band observed at 1504 cm^−1^. The %PED of this stretching mode by CAM-B3LYP (52%) differs from that with B3LYP (18%). This assignment finds further support in the experimental IR band observed at 1510 cm^−1^ in 5-chlorocytosine and 5-bromocytosine [2], and in the experimental Raman line of these molecules, at 1512 and 1495 cm^−1^, respectively, in good accordance with our results.

Another lower contribution of ν(C-N4) identified in mode 21 (ν_21_) was related to the strongest IR band observed experimentally at 1302 cm^−1^, and the very strong Raman line at 1301 cm^−1^, although in our tetramer form calculations, they appear with medium IR and Raman intensities. This experimental assignment is in accordance with that found at 1285 cm^−1^ (IR) and at 1310 cm^−1^ (Raman) in 5-bromocytosine [2].

**N1-H modes:** The N1-H and CH stretching modes usually appear in the 3200–3000 cm^−1^ range with the N1-H mode on a higher frequency side. In 2TC, ν(N1-H) (ν_32_) was predicted (scaled) with B3LYP at 3112 cm^−1^ with strong IR intensity, and at 3110 cm^−1^ with medium Raman intensity, in excellent accordance with the very strong IR band at 3110 cm^−1^ and medium Raman line at 3109 cm^−1^. The δ(N1-H) in-plane bending (ν_20_) was scaled at 1242 cm^−1^ with B3LYP and at 1234 cm^−1^ with CAM-B3LYP, in good accordance with the experimental IR band at 1235 cm^−1^ and the Raman line at 1249 cm^−1^. The scaled value at 1217 cm^−1^ (with B3LYP) and 1206 cm^−1^ (with CAM-B3LYP) corresponds to N1-H groups, which are not involved in H-bonds, i.e., free groups, and the strong IR band detected in the experimental spectrum at 1200 cm^−1^ can be correlated well with this mode.

The out-of-plane bending γ(N1-H) (ν_10_) appears as almost pure mode (87% PED), and in H-bonded groups, it is scaled at 902 cm^−1^ (with B3LYP) and 906 cm^−1^ (with CAM-B3LYP) and related to the IR band at 930 cm^−1^ and the Raman line at 932 cm^−1^. In free N1-H groups, it is scaled at ca. 640 cm^−1^ and tentatively related to the strong IR band observed at 652 cm^−1^.

**C-S modes:** The main contributions of the stretching ν(C-S) mode were identified in the scaled wavenumber at 1109 cm^−1^ with B3LYP and are correlated well with the strong IR band at 1098 cm^−1^ and in the scaled value at 717 cm^−1^ and related to the IR band at 724 cm^−1^ and the Raman line at 718 cm^−1^. Small contributions lower than 10%PED of this stretching mode were identified in the neighbouring scaled wavenumbers at 1171, 970 and 932 cm^−1^. The C=O group gives rise to a prominent and characteristic stretching band, but the C-S group of 2TC does not show this prominence. Our assignment is in accordance with that found at 1143 cm^−1^ in the tetramer form of 2TU [57] and corresponds to the experimental IR/Raman band at 1160 cm^−1^ and is also in further agreement with the literature value [30].

A small contribution of the in-plane bending δ(C-S) mode was identified in 2TC at 451 cm^−1^, in good agreement with the IR band at 456 cm^−1^ and the Raman line at 452 cm^−1^. This assignment finds further support in the scaled values at 464 (IR) and 463 (Raman) cm^−1^ reported in 2TU and their corresponding bands at 454 (IR) and 430 cm^−1^ (Raman) [57]. Another small contribution of this δ(C-S) mode was identified in the scaled wavenumber at 203 cm^−1^ and related to the experimental bands at 214 (IR) and 215 cm^−1^ (Raman). It is noted that in the uracil molecule, the wavenumber of the δ(C-S) mode remarkably changed by ~100 cm^−1^ when the sulphur substitution was at C2, but this change was remarkably lower when it was at the C4 position [58].

The out-of-plane bending γ(C-S) mode was scaled at 667 cm^−1^ with 30%PED and related to the IR band at 652 cm^−1^ and the Raman line at 659 cm^−1^. Small contributions were also identified in the scaled values at 436 and 203 cm^−1^, in excellent agreement with the experimental IR bands at 434 and 214 cm^−1^, respectively. This assignment finds support in the scaled wavenumber at 175 cm^−1^ reported in the tetramer form of 2TU and related to the Raman line at 138 cm^−1^. It is also in accordance with the 130–160 cm^−1^ spectral range [59] reported for this mode and in literature values in similar molecules [57].

### 2.5. Hydration of 2TC

Solvation has a dramatic influence on the tautomerism of cytosine. Polar solvents stabilize the keto T1 form over the enol T2b form. This feature greatly reduces the chance of seeing this tautomer in biomolecules, which is not surprising, as living systems need to remove rare tautomers in most situations. Therefore, in many theoretical reports [60,61,62] nucleobase hydration has been analysed. For that, two procedures have been mainly recommended for this theoretical task: (i) the continuum model (CM) utilising diverse solvation cavity techniques and (ii) the discrete model (DM). In our previous calculations carried out in related molecules, this DM model with a sufficient explicit number of water molecules surrounding the nucleobase was found to be the best [57], and therefore, it was used in the present study. The specific procedure followed with this model for hydration was the so-called Modified Scheme of Monosolvation (MSM) [22].

**Figure 11:**
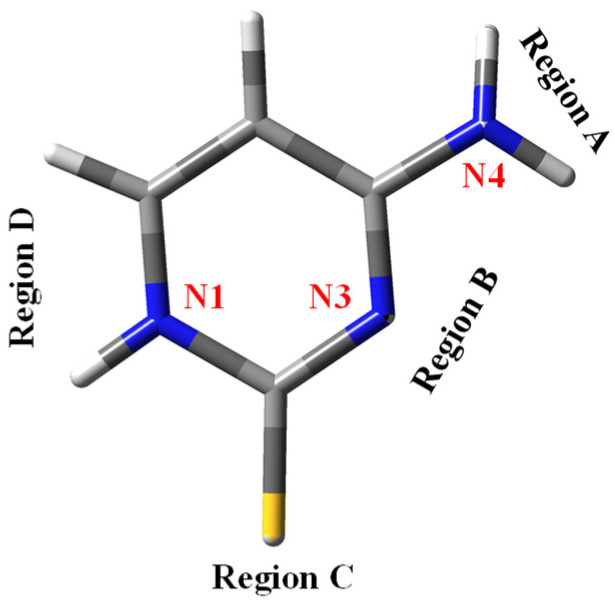
Representation of the four regions available for hydration in 2TC.

Four regions appear available for hydration in 2TC (Figure 11). Region A is with the –NH_2_ group H-bonded to two water molecules, each one to each amino hydrogen. In B and D regions, only one water molecule can be H-bonded but in a stronger way than in region A, especially on N1 which has a higher negative charge than on N3. Region C is the most appropriate for hydration in cytosine molecules with strong H-bonds through the O2 oxygen atom, but it is less favourable in 2TC because of the weak H-bonds with the sulphur atom, where only two water molecules can be bonded weakly.

Because the attractive water–water interaction appears stronger than between water-2TC, ‘clustering’ structures of these water molecules always appear more stable than ‘distributed’ structures with water molecules surrounding 2TC (or cytosine) molecules. We also noted this feature in related nucleobases [57]. However, our interest is in ‘distributed’ structures to know the hydration effect better on the tautomerism and molecular structure of 2TC.

**Figure 12:**
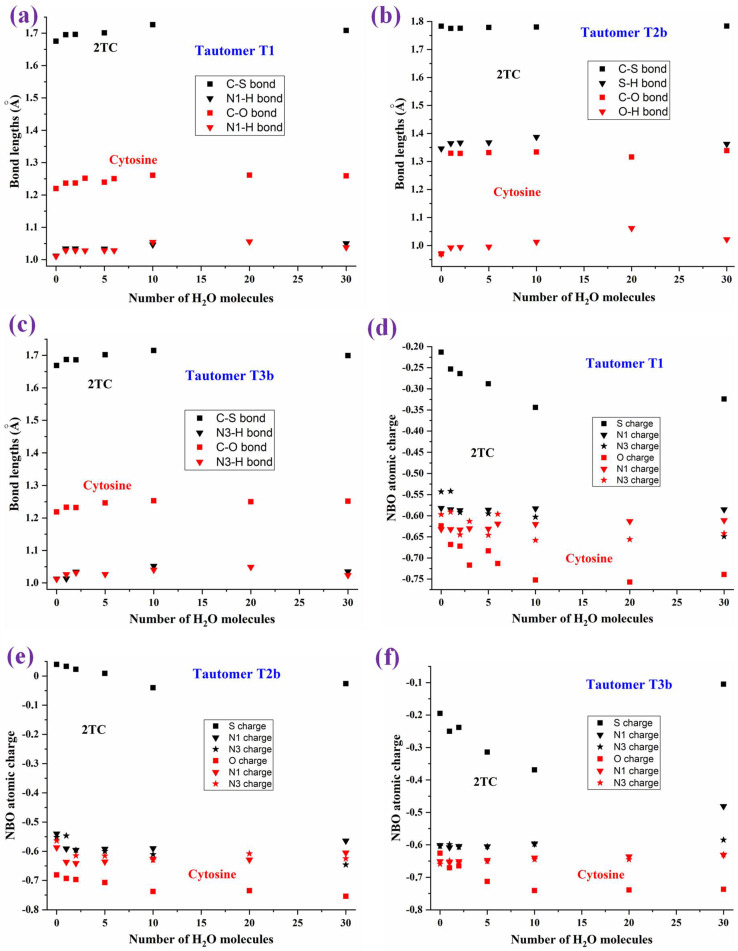
Comparison of several selected bond lengths and NBO atomic charges in the three most stable tautomers of 2TC and cytosine with hydration up to 30 water molecules. (**a**) Variation of the N1-H and C-S (or C=O with cytosine) bond lengths in the different clusters with the keto tautomer T1. (**b**) Variation of the N1-H and C-S (C=O) bond lengths in the enol tautomer T2b. (**c**) Variation of the N1-H and C-S (C=O) bond lengths in the imino tautomer T2b. (**d**) Variation of the NBO atomic charge on N1, N3, and S (or O with cytosine) atoms in the different clusters with the keto tautomer T1. (**e**) Variation of the NBO atomic charge on N1, N3, and S (or O) atoms in tautomer T2b. (**f**) Variation of the NBO atomic charge on N1, N3, and S (or O) atoms in tautomer T3b.

**Figure 13:**
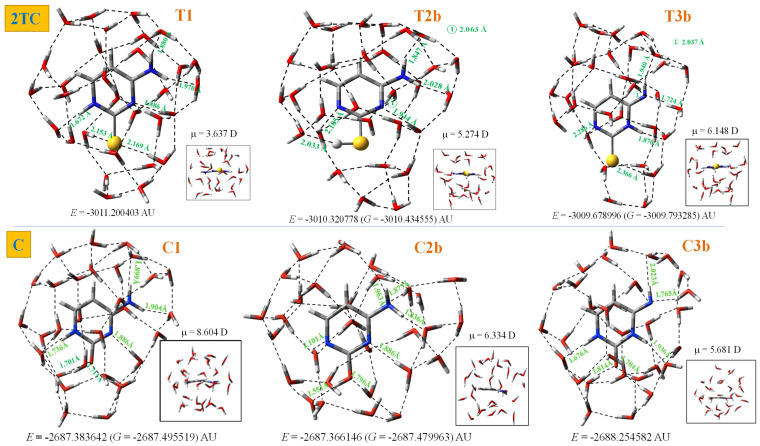
Comparison at the B3LYP/6-31G(d,p) level of the optimised hydrated clusters with 30 water molecules in tautomers T1, T2b, and T3b of 2TC with those of the cytosine molecule, which include the first and second hydration shells. Two views of each cluster are included, together with the total energy of each cluster and its dipole moment.

The hydration effect on 2TC tautomerism was determined under the DM model up to 30 explicit water molecules, which includes the estimated water molecules range for the first and second hydration shells (Figure 12 and Figure 13). The geometrical structures of these clusters are expected to be close to that in bulk water. Only the hydration of **T1**, **T2b,** and **T3b** tautomers in 2TC was considered and compared to the related ones in the cytosine molecule, with these tautomers labelled as C1, C2b, and C3b for convenience. For simplicity, the clusters obtained with the hydration progress following the MSM scheme were not included, and only the final results with 30 water molecules were plotted and discussed. Two views of each optimised cluster (front and perpendicular forms) were represented. The intermolecular H-bond values of the water molecules directly bonded to 2TC were also included, together with the corresponding total energy of the system and its dipole moment value (bottom of each figure).

The first hydration shell was fully obtained with ten water molecules, with six of them appearing to be directly H-bonded to the nucleobase. The second hydration shell was reproduced well by 20 water molecules. Therefore, the results shown with 30 water molecules almost represent the third hydration shell and the bulk. Through these clusters, the hydration effect in the N1-H and C-S (or C=O with cytosine) bond lengths can be observed in Figure 12a, which corresponds to the T1 tautomer. Hydration causes bond length alteration with a lengthening of the C-S and N1-H-bonds and consequently shortening of the bond lengths adjacent to it, as well as an opening of the *ipso* angles involved in H-bonds. At the beginning of hydration, this lengthening is remarkably observed with every water molecule that is added, although with more than 10 water molecules, lengthening is remarkably reduced and the value remains almost constant with further water molecule addition. A similar trend was observed with the cytosine molecule, which is included in this figure for comparison purposes.

The hydration effect in the C-S and S-H bond lengths of the T2b tautomer is included in Figure 12b, while the effect in the C-S and N3-H-bonds of the imino T3b tautomer is plotted in Figure 12c. Similar lengthening of the bond lengths at the beginning of hydration is observed in comparison with the cytosine molecule.

The hydration effect on the S, N1 and N3 NBO atomic charges is plotted in Figure 12d–f. A sharp change in the sulphur charge is observed with the increasing number of water molecules from the isolated state to 10 water molecules in the three tautomers, and then it increases a little with further hydration. The sharp decrease is lower in the oxygen atom. The hydration effect on N1 and N3 atoms is lower and does not follow a clear trend.

The other main features observed with hydration were the following:

(i) With hydration progression, the net water molecules in 2TC differ somewhat from those of the cytosine molecule due to lower attraction of S···H_2_O than O···H_2_O.
(ii) Interaction between the 2TC tautomers and one water molecule decreases the difference in the energies of the two most stable tautomers significantly, stabilising the **T2b** (**T2a**) forms more than the **T1** one, but it does not change the stability order of the tautomers [25]. However, in the first hydration shell with six water molecules, the stability pattern changes, although the **T1** form cluster remains the most stable one. The heights of the barriers of sulphur-involved proton transfers in 2TC are much smaller than analogous barriers in cytosine [25].
(iii) With the exception of the weak H-bonds surrounding the C-S moiety, the H-bonding net around 2TC is similar to that in cytosine in all of the clusters. This feature is in accordance with that found in comparisons of other thiobases with their parent oxomolecules [63]. Although the net is similar, the negative NBO charge on N1 and N3 atoms appear remarkably smaller in 2TC than in cytosine, leading to N···H_2_O H-bonds with the water molecules weaker than the corresponding ones in cytosine. However, the far C5 and C6 atoms are not affected by this feature.
(iv) With hydration progression, the intermolecular H-bonds through the sulphur atom are remarkably stretched.
(v) The water molecules only slightly affect the large planarity of the pyrimidine ring, with a change in its torsional angles of less than 2°. However, lengthening of the N-H-bonds and opening of the ipso ring angles involved in H-bonds were observed.
(vi) With hydration, the NH_2_ group appears tilted ~ 1° out of the C4-C5-C6 plane. This small deformation is due to its large flexibility, with it being greater in 2TC than in cytosine. Large flexibility facilitates easier geometry relaxation in 2TC to form more appropriate intermolecular interactions with other molecules.
(vii) The hydration effect on bond lengths decreases until the first hydration shell, remaining almost unaffected with further water addition. The changes in the torsional angles, in general, lower than 10°, indicate large ring flexibility. An increment in this flexibility appears with a decrease in the resonant form contribution to the total structure [64].
(viii) Water molecule distribution changes the μ values considerably. With hydration progression, its value changes irregularly owing to the large water polarity. However, it noticeably increased with more than 20 water molecules.

### 2.6. Watson–Crick and Reverse WC Pair and Differences with Canonical Ones

#### 2.6.1. Biological Importance

In canonical B-DNA, cytosine (C) pairs with guanine (G) through three H-bonds, providing considerable DNA stability. Therefore, the C:G base pairs in genomic DNA appear to correspond to recognition sites, including transcription initiation. However, some pathogens, such as the Mycobacterium tuberculosis bacteria, also contain C:G-rich sequences in their genome. This has been interpreted as due to the lack of nutrition and the high-stress conditions, where these bacteria are required to survive a large number of C:G pairs to provide greater stability to their genome. Modifications or alterations in the base pair scheme owing to a tautomeric form can lead to a reduction in DNA stability. The presence of a thio-substituted base may be even more harmful to DNA stability. Thus, the search for the most stable tautomer and the stability of its formed WC pair can provide important information on DNA structures containing substituted nucleobases.

In the uracil molecule, replacing sulphur on any of the carbonyl oxygen molecules increases the nucleotide’s ability to inhibit orotidine-5′-monophosphate decarboxylase (ODCase) [65], and a similar feature is expected with 2TC. In addition, molecular docking studies of 2TC against different COVID-19 receptors indicate that it can be considered as a better antiviral agent [3]. The effect of the sulphur atom in thiobases on the DNA helix has been little studied at the molecular level [57,63]. Therefore, to shed some light on this matter, firstly, the WC pairs with 2TC were analysed.

#### 2.6.2. Optimised WC Pairs with 2TC

**Figure 14:**
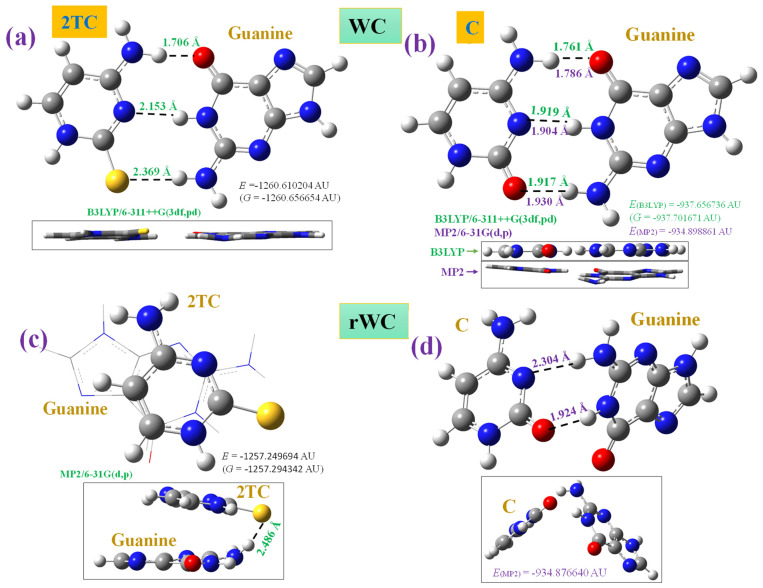
Optimised WC pairs of (**a**) 2TC with guanine in *cis* form (canonical form) at the B3LYP/6-311++G(3df,pd) level; (**b**) cytosine with guanine in *cis* form (canonical form) at the B3LYP/6-311++G(3df,pd) and MP2/6-31G(d,p) levels; (**c**) 2TC with guanine in *trans* form (reverse form) at the MP2/6-31G(d,p) level; (**d**) cytosine with guanine in *trans* form (reverse form) at the MP2/6-31G(d,p) level.

The sulphur atom effect in the 2TC:G WC pair, the cis form (Figure 14a), was analysed through comparison with the C:G pair (Figure 14b). For this task, both pairs were optimised first at the B3LYP/6-311++G(3df,pd) level, and in addition, the C:G pair was also optimised at the MP2/6-31G(d,p) level. The values of the intermolecular H-bond lengths were included in these figures, as well as the total energy + ZPE correction obtained for the system and the Gibbs energy. All optimised structures show positive wavenumbers, i.e., they are stationary points. The green color was used for B3LYP results and the violet color was used for MP2. Front and perpendicular views were shown for each figure. An almost planar form was obtained through the calculations with B3LYP, but slightly out-of-planarity appeared between C and G nucleobases in the calculation with MP2. One of the reasons is due to the pyramidalization of the NH_2_ group being much higher with MP2 than with B3LYP, and because of this NH_2_ group being involved in the intermolecular H-bonds, this leads to a slight rotation of the G nucleobase.

The sulphur atom effect was also studied in the reverse *trans* form (rWC) of the 2TC:G pair (Figure 14c) and compared to the reverse C:G pair, Figure 14d. The calculations were carried out with the MP2, B3LYP, and CAM-B3LYP methods for 2TC. Owing to the involvement of the sulphur atom in weaker hydrogen bonds with G in the WC pair, with longer S···H-N bonds (Figure 14c), this facilitates greater flexibility of the pair to form a new stacking shape with increased π-π type interactions between the nucleobases.

The rWC C:G pair also shows a perpendicularity between C and G nucleobases, but their initial intermolecular H-bonds were not broken, and the nucleobase rotation facilitates these H-bonds through the pyramidalization of the NH_2_ group. Although this reverse form does not appear to be as stable from a structural point of view, it is known to participate in the interaction between the D-loop and the V-loop in tRNA structures.

The molecular geometry of canonical (Figure 14b) and sulphur-substituted (Figure 14a) WC pairs show some similarity, but in the rWC pair, they differ noticeably. Thus, in the WC form, 2TC has three intermolecular hydrogen bonds with G through the amino groups and sulphur atom. This pair appears more open towards the minor groove side than the cytosine pair (Figure 14b). This is due to the large differences between C-S···H-N and C=O···H-N hydrogen bonds and the corresponding large lengthening of both C-S (or C=O) and N-H bonds. Other differences are the following:

(i) Shortening of the N-H bond length in guanine when the H-bond is with S instead of with oxygen.
(ii) Lengthening of the C-S bond, 1.657 Å, related to C=O, 1.226 Å in the isolated state at the MP2/6-31G(d,p) level. This feature leads to noticeable lengthening of the intermolecular H-bond C-S···H-N(G), 2.369 Å vs. 1.917 Å in C=O···H-N(G) in the C:G pair, and also lengthening of N3···H-N(G), 2.153 Å vs. 1.919 Å in the C:G pair, and shortening of the N-H···O(G), 1.706 Å vs. 1.761 Å in the C:G pair.
(iii) In the rWC pair, there are larger changes with 2TC than with cytosine, with only one intermolecular H-bond of the two possible bonds with the MP2 calculation. Since reverse forms are present in tRNA, stacking interactions can therefore be responsible for their stabilisation.
(iv) The calculated interaction energies are higher in the C:G pair than 2TC:G and higher than those in the rWC pair, Table 3, as expected. The deformation energy E^def^ is a little lower in 2TC than in cytosine, and this energy appears higher in guanine interacting with cytosine than with 2TC. The calculated lower stability of the 2TC:G pair (ΔE^CP^ = −97.458 kJ/mol) than the C:G pair (−105.930 kJ/mol) is in accordance to the reported value of 8 kJ/mol [63].
(v) The calculated μ value in the 2TC:G pair, 4.205 D, is lower than in C:G, 5.793 D, in contrast with that found in thiouracils, where the thio group increases the polarizability and μ [63].
(vi) The computed entropy in the 2TC:G pair is a little higher (134.33 J/mol·K) than in the C:G pair (130.14 J/mol·K).

### 2.7. Sulphur Atom Effect in Optimised DNA:DNA Microhelices

**Figure 15:**
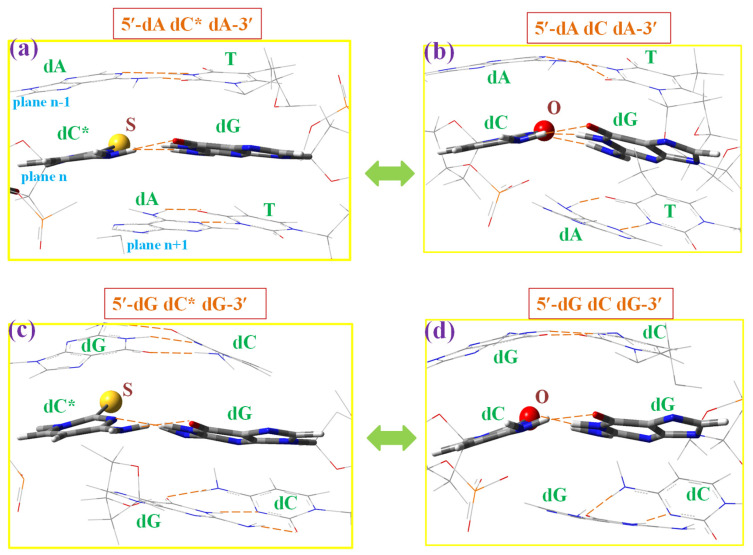
A perpendicular view of the intermolecular H-bonds in the optimised (**a**,**c**) 5′-dA dC* dA-3′ and 5′-dG dC* dG-3′ microhelices with 2TC (**b**,**d**) compared to the corresponding canonical ones with cytosine.

**Figure 16:**
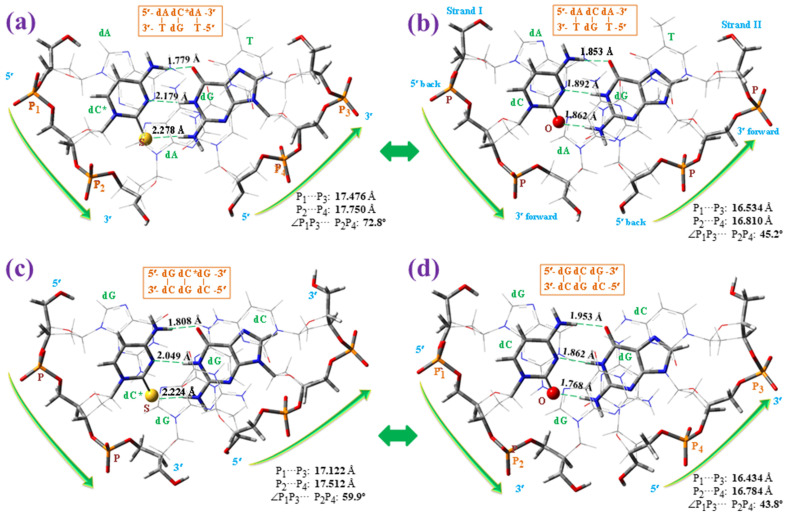
Other view of the intermolecular H-bonds in the optimised (**a**,**c**) 5′-dA dC* dA-3′ and 5′-dG dC* dG-3′ microhelices with 2TC (**b**,**d**) compared to the corresponding canonical ones with cytosine.

**Figure 17:**
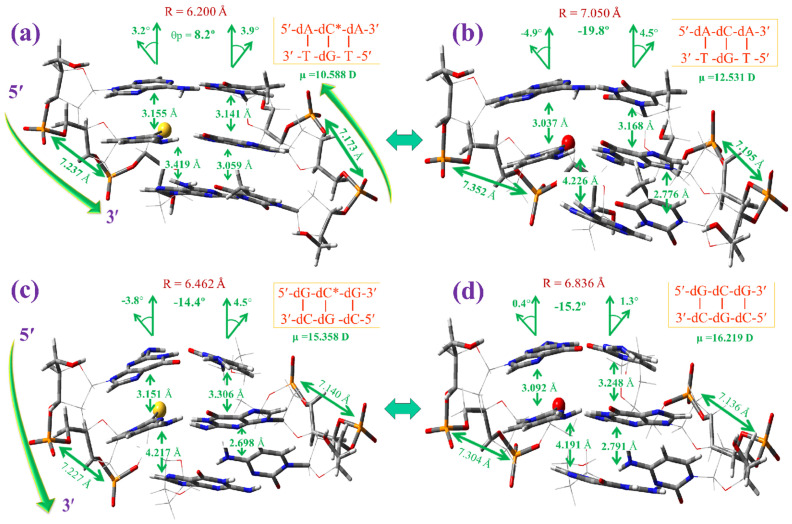
Effect of the sulphur atom of 2TC on the helical parameters of the: (**a**,**b**) 5′-dA dC* dA-3′ microhelix compared to canonical one 5′-dA dC dA-3′. (**c**,**d**) 5′-dG dC* dG-3′ microhelix compared to canonical one 5′-dG dC dG-3′.

Although 2TC appears to be present in tRNA, a first and short study about the possibility of its use as an antiviral or anticancer drug by breaking or deforming the DNA helix has been carried out herein, as reported in the case of the 2-thiouracil molecule [66]. For this purpose, a simplified system of the DNA–DNA helix was optimised at the M06-2X/6–31G(d,p) level with the three nucleotide base pairs. Only the sulphur atom effect in the 5′-dA-dC-dA-3′ and 5′-dG-dC-dG-3′ microhelices of the B-type was analysed. The notation used for the microhelices was the same as reported in our previous works [66], with a short form using only the nucleosides of strand *I* (Figure 15) and with a full form using the nucleosides of both strands (Figure 16 and Figure 17). For simplicity, the notation dC* was used for 2TC. The nucleoside with 2TC was placed in the central plane *n* of the microhelix. Therefore, the effect of 2′-deoxyadenine (dA) in the up and down planes, *n* − 1 and *n* + 1 planes, respectively, can be analysed in the 5′-dA-dC*-dA-3′ microhelix with 2TC, and compared to the canonical one with cytosine, 5′-dA-dC-dA-3′. The effect of 2′-deoxyguanosine in the up and down planes of the 5′-dG-dC*-dG-3′ microhelix was also studied. The B-type helix notation corresponds to double-helices with the furanose ring in the C_2′_-*endo* orientation [11]. The B-type is the most common in DNA helices, while the A-type (C_3′_-*endo*) appears in RNA and hybrid helices.

Several rotational and translational parameters are reported to explain the geometric relations between bases and base pairs [11,67], but only the three most important ones are analysed herein: (i) The rise parameter (Dz) corresponding to the distance between bases in the stacking form, (ii) the helix radius (R), and (iii) the propeller twist θp, which is the dihedral angle between individual base planes.

Figure 15 shows a comparison between the microhelices with sulphur (Figure 15a,c) and those with oxygen, canonical ones (Figure 15b,d). Only the intermolecular H-bonds between both strands of the helix is shown in these figures. In the helix, the dC*:dG pair with 2TC appears with a slight rotation in opposite to the almost full planar form found in the base pair alone, Figure 14a. This rotation appears slightly larger in the microhelices with the canonical dC:dG pair. The planarity with dA in the planes *n* − 1 and *n* + 1, Figure 15a,b is slightly higher than with dG (Figure 15c,d). Compared to canonical microhelices, the effect of the sulphur atom appears stronger higher in microhelices with dA than with dG.

Figure 16 shows a small comparison of the microhelices from the *z*-axis (helix-axis). The base pair atoms in plane *n* are marked with sticks, as well as the helix backbone. Upon comparing the values in these microhelices with those of the base pair alone, the following was observed:

(i) The cytosine ring is whole planar in cytosine and 2TC molecules, but it appears slightly out-of-plane in the helix. A similar feature has been found with uracil and its derivatives [66]. This out-of-planarity in the base pair of the helix has been interpreted as a result of the change in χ angle by the phosphate bond torsion [61].
(ii) The long S···H-N(G) intermolecular H-bond in the base pair alone is shortened in the helix of Figure 16a, while N3···H-N(G) and N4-H···O(G) bonds are lengthened. In Figure 16c, the S···H-N(G) and N3···H-N(G) bonds are shortened, while N4-H···O(G) is lengthened. This average of the values is also observed in the canonical WC pair, with shortening of the O···H-N(G) and N3···H-N(G) long bonds and lengthening of the N4-H···O(G) short bond (Figure 16b,d).
(iii) The shift in the values from (base pair alone) → helix is ca. twice as high in the 5′-dG-dC*-dG-3′ microhelix than in 5′-dA-dC*-dA-3′.

Figure 17 shows the microhelices of Figure 16 but from the *y*-axis. Several characteristic helical values were also included. Comparing the optimised parameters in these microhelices, the following was observed:

(a) Owing to the base pair stability with sulphur being lower than with oxygen, the exocyclic torsional angles χ, ζ, α, β and γ [66] of the helix appear considerably greater with 2TC than the related ones with cytosine, which leads to remarkable changes in the microhelices with 2TC.
(b) The distance between the phosphorus atoms in each strand is slightly shorter in the 5′-dG-dC*-dG-3′ microhelix than in 5′-dA-dC*-dA-3′, which can be explained by interplanar π-π interactions of cytosine with guanine in the *n* − 1 and *n* + 1 planes being higher than cytosine with adenine in the 5′-dA-dC*-dA-3′ microhelix. This distance between the phosphorus atoms is also shorter in the microhelices with 2TC than the related ones with cytosine.
(c) The microhelix radius (R) with 2TC appears considerably shorter than the canonical ones with cytosine. Due to this feature, intermolecular H-bonds between both strands of the helix are weaker with 2TC than with cytosine.
(d) The propeller twist angle θp is lower in the microhelices with the sulphur atom, especially in 5′-dA-dC*-dA-3′.
(e) The dipole moment μ is lower in the microhelices with 2TC, due to the lower electronegativity of the sulphur atom than the oxygen atom of cytosine. The μ value is considerably larger with 5′-dG-dC*-dG-3′ than with 5′-dA-dC*-dA-3′.

Although the sulphur atom in 2TC leads to noticeable changes in the helical parameters, the optimised microhelices appear stable and can be present in biological media.

## 3. Materials and Methods

The computations were mainly performed with B3LYP, CAM-B3LYP, and M06-2X DFT functionals [68] using the GAUSSIAN16 package [69] in the Brigit super-computer of Complutense University of Madrid. Density functional theory (DFT) methods are commonly used today because they present an excellent description of medium-size molecules, especially for wavenumber calculations in biomolecules [1,69,70,71,72].

Among the DFT methods, the Minnesota M06-2X functional was also chosen because it appears as one of the best meta-generalized gradient functionals to analyze dispersion–bound in large systems [73,74], especially in biomolecules with non-covalent weak interactions. Thus, the DNA–DNA microhelices that include the 2TC nucleobase were optimised with this method and the 6-31G(d,p) basis set. This method has also shown large applicability in chemistry [66,75]. Previous calculations with the B3LYP method [76,77] appear to fail in the description of the DNA:RNA microhelix [57], as well as with M052X [78] and LC-wPBE (long-range-corrected version of wPBE [79]) with a remarkably deformed microhelix, and thus, they were not used here.

However, for vibrational calculations, the CAM-B3LYP method [80], which combines the hybrid qualities of B3LYP and the long-range correction presented by Tawada et al. [81] was used to improve the calculated structural parameters and vibrational wavenumbers. The B3LYP method was also used because it shows a good description of medium-size molecules and large accuracy in wavenumber computations [54], and for this reason, it is the most popular among DFT methods and it was mainly used in the present study for the interpretation of the experimental spectra. Moreover, the accuracy obtained in the wavenumber calculation is noticeably higher than with M06-2X [54]. Several electronic properties were computed with these methods. HF and MP2 are more costly computational methods and dramatically fail in this type of calculation [46,52,53,54].

MP2 [82] and CCSD ab initio calculations were carried out to obtain a better relative stability trend with the different tautomers and to compare it with those obtained in cytosine. MP2 calculations were also used in one of the tetramer forms to finally confirm its molecular structure. These methods appear to have an excellent accuracy/computational cost ratio for this type of calculation.

### Interaction Energy Calculation in 2TC Molecule

The interaction energies (E^int^) were determined only in the planar and non-planar tetramer forms with the **T1** tautomer of 2TC and cytosine molecules at the B3LYP/6-311++G(3df,pd) and CAM-B3LYP/6-311++G(3df,pd) levels because they are the expected forms in the solid-state crystal. These were also calculated in the 2TC:G and C:G WC pair in planar form with T1 tautomer, and therefore the effect of the sulphur atom in the pair can be determined through comparison. All calculated energies have been corrected with the Boys and Bernardi procedure for basis set superposition error [83,84]. Thus, the total counterpoise (CP) corrected interaction energy, $\Delta E_{\mathrm{AB}}^{\mathrm{CP}}$, in a system (AB) were determined using following the relationship:

(1) $$\Delta E_{\mathrm{AB}}^{\mathrm{CP}}=E^{\mathrm{int}}(\mathrm{AB})+E^{\mathrm{def}}(\mathrm{AB})$$

where (AB) corresponds to all molecules of the system, which differs depending on the system considered. In the present study, the equations used to determine E^int^(AB) were the following:

(i) In the tetramer form of 2TC and cytosine molecules, (AB) corresponds to the four tetramer molecules, labelled as A, B, Cm and D. The interaction energy E^int^(AB) between these molecules is given as Equation (2) below:
  (2) $$E^{\mathrm{int}}(\mathrm{AB})=E_{AB}^{AB}(\mathrm{AB})-E_{A}^{AB}(\mathrm{AB})-E_{B}^{AB}(\mathrm{AB})-E_{C}^{AB}\left(\mathrm{AB})-E_{D}^{AB}(\mathrm{AB}\right)$$
  where $E_{AB}^{AB}$(AB) is the electronic energy of the tetramer form (AB), and $E_{A}^{AB}$(AB) is the electronic energy of monomer A in this tetramer (AB), and $E_{B}^{AB}$ corresponds to monomer B, and so on with each monomer.
(ii) In the WC base pairs of 2TC and cytosine molecules, the interaction energy E^int^(AB) is between both molecules (AB) of the base pair, which in 2TC:G corresponds to molecule A (≡2TC) and B (≡guanine), and in the natural WC pair, molecule A (≡cytosine) and B (≡guanine). Therefore:
  (3) $$E^{\mathrm{int}}(\mathrm{AB})=E_{AB}^{AB}(\mathrm{AB})-E_{A}^{AB}\left(\mathrm{AB})-E_{B}^{AB}(\mathrm{AB}\right)$$
  where $E_{AB}^{AB}$(AB) is the electronic energy of the WC base pair (AB), and $E_{A}^{AB}$(AB) the electronic energy of molecule A in this base pair (AB).

The total deformation energy E^def^(AB) was determined as the sum of the deformation energies of each molecule in the system:

(i) In the tetramer form of 2TC and cytosine molecules, E^def^(AB) can be written as E^def^(AD) related to the four tetramer molecules (A, B, C, and D), thus obtaining the following equation:
  (4) $$E^{\mathrm{def}}(\mathrm{AD})=E_{A}^{\mathrm{def}}(\mathrm{AD})+E_{B}^{\mathrm{def}}(\mathrm{AD})+E_{C}^{\mathrm{def}}(\mathrm{AD})+E_{D}^{\mathrm{def}}$$
  where $E_{A}^{\mathrm{def}}$ is the deformation of monomer A inside the tetramer (AD) that was determined for monomer A, as follows, but it can be applied to every monomer of this tetramer:
  (5) $$E_{A}^{\mathrm{def}}(\mathrm{AD})=E_{A}^{A}\left(\mathrm{AD})-E_{A}^{A}(A\right)$$
  where the parentheses indicate whether the computations were performed at the optimised geometry of the monomer (A) or tetramer (AD). Superscripts show that the calculations were performed with the basis set of monomer (A), and the subscript indicates monomer (A).
(ii) In the WC base pairs, E^def^(AB) corresponds to the following equation:
  (6) $$E^{\mathrm{def}}(\mathrm{AB})=E_{A}^{\mathrm{def}}(\mathrm{AB})+E_{B}^{\mathrm{def}}$$
  where $E_{A}^{\mathrm{def}}$ or $E_{B}^{\mathrm{def}}$ indicate each molecule of the pair and were determined as:
  (7) $$E_{X}^{\mathrm{def}}(\mathrm{AB})=E_{X}^{X}\left(\mathrm{AB})-E_{X}^{X}(X\right)$$
  where X (≡A or B), and the parentheses indicate whether the computations were performed at the optimised geometry of each monomer (A or B) or the pair (AB). The superscript indicates that the calculations were performed with the basis set of monomer (X), and the subscript specifies the monomer considered.

## 4. Summary and Conclusions

A detailed study on 2TC biomolecules in gas-phase, solid-state, and hydrated forms was carried out from the structural and spectroscopy points of view. Its WC interactions as nucleobases in a DNA–DNA microhelix was also considered. The most important findings of the present study were the following:

(1) The sulphur atom does not show any effect on the tautomeric stability trend with the CCSD(T) method. Tautomer **T2b** is the most stable one and slightly more stable than **T2a**. Both tautomers have the possibility of co-existence in the gas phase, due to the energy difference between both being even twice as low in 2TC than in cytosine. The *keto* tautomer **T1** in 2TC is almost four times less stable than that in cytosine.
(2) The scaled IR spectrum of the **T2b** tautomer is in excellent accordance with that found experimentally in the Ar and N_2_ matrices, indicating that only **T2b** (and **T2a**) tautomers exist.
(3) The scaled wavenumbers in the planar, non-planar, and stacking tetramer forms were determined at different DFT levels. The results obtained with these tetramers seem more in agreement with the experimental IR and Raman spectra in the solid state than with those previously reported in the monomeric form. The concordance obtained in the planar tetramer form indicated that only the **T1** tautomer exists in the solid state.
(4) The use of an accurate scaling equation procedure remarkably reduces the error in the calculated wavenumbers. Thus, the assignment of most of the fundamentals provided in this study is believed to be unambiguous. These assignments were based on all 33 modes identified and compared to the cytosine molecule.
(5) Clusters of **T1**, **T2b**, and **T3b** tautomers with explicit water molecules up to 30 were optimised in 2TC and cytosine molecules for the first time, which includes the expected range of water molecules for the first and second hydration shells. The water molecules only slightly affect the planarity of the pyrimidine ring, with changes in its torsional angles of less than 2°. The attraction forces between the water molecules and sulphur atom are lower than with the water molecules and the oxygen atom of cytosine, which leads to a water distribution around the sulphur atom clearly different of that observed with cytosine oxygen in all studied tautomers. This different water distribution changes the tautomer stability, as well as their properties, with a clearly different dipole moment. Hydration confirms that the **T1** *keto* tautomer form is the most stable one.
(6) Hydration increases the negative NBO charge on the oxygen atom in the cytosine molecule and slightly on the sulphur atom of the **T1** tautomer of 2TC and decreases on nitrogen atoms, leading to higher HOMO and LUMO orbital energies.
(7) The canonical WC pairs with guanine appear planar, while the reverses are out-of-plane. The sulphur atom leads to base pair destabilisation and the reverse form appears in the stacking arrangement with MP2. The pair with 2TC is slightly more open toward the minor groove side than the cytosine pair and more rotated. E^int^ is higher in the C:G pair than 2TC:G, because E^def^ is higher in cytosine than in 2TC.
(8) The pyrimidine ring of 2TC and cytosine appears slightly out-of-plane in the helix. The distance between the phosphorus atoms in each strand is slightly shorter in the 5′-dG-dC*-dG-3′ microhelix than in 5′-dA-dC*-dA-3′.
(9) The intermolecular H-bonds between both strands of the helix appear weaker with 2TC than with cytosine, which leads to the P···P phosphorus distance in each strand being shorter in the microhelices with 2TC than in the canonical ones. With 2TC, it also leads to a shorter rise parameter, a shorter helix radius (R), and a lower θp angle. All of these features contribute to helix deformation in the microhelices with 2TC and therefore, it may be used as an anticancer drug.

## Figures and Tables

**Table 1:** Gas-phase relative energies (kJ/mol) of 2TC and cytosine tautomers.

Methods	T1	T2a	T2b	T3a	T3b	T4
**Cytosine:**	5.8	2.9				
MP2(full)/6-311+G(2d,2p) ^a^ + ZPE	5.9	3.1	0.0	20.6	13.3	37.4
CCSD(T)//MP2/cc-pvtz(-f) ^b^	6.1	2.8	0.0	13.7	7.5	-
MP2/6-31+G(d,p) + ZPE	5.32	3.00	0.0	17.44	10.76	35.22
ΔG	3.81	2.97	0.0	16.82	10.10	33.34
CCSD(T)/6-31+G(d,p)//MP2/6-31+G(d,p)	5.92	2.96	0.0	16.64	9.83	36.22
**2TC:**						
MP2/6-31+G(d,p) + ZPE	25.72	1.31	0.0	37.34	30.90	55.90
ΔG	25.16	1.27	0.0	38.00	31.50	54.76
CCSD(T)/6-31+G(d,p)//MP2/6-31+G(d,p)	16.94	1.24	0.0	27.00	20.38	47.52

^a^ //MP2(full)/6-31G*, ref. [44]. ^b^ Ref. [45].

**Table 2:** Optimised geometrical parameters at the B3LYP/6-311++G(3df,pd), CAM-B3LYP/6-311++G(3df,pd), and MP2/6-31+G(d,p) levels in the tetramer form of 2TC with tautomer T1, as compared to the experimental X-ray data. The values corresponding to the second molecule of the system (molecule B, Figure 3a) are shown in parentheses, and those without them correspond to molecule A.

	MP2	B3LYP		CAM-B3LYP	MP2	X-Ray
Parameters	Isolated State	Planar	Non-Planar	Planar	Non-Planar	A (B)
*Bond lengths (Å)*						
N1-C2	1.402	1.391 (1.383)	1.389 (1.381)	1.381 (1.373)	1.387 (1.378)	1.369 (1.367)
C2-N3	1.371	1.343 (1.342)	1.344 (1.340)	1.338 (1.336)	1.356 (1.361)	1.342 (1.343)
N3=C4	1.325	1.344 (1.327 *)	1.343 (1.329 *)	1.338 (1.321)	1.346 (1.330)	1.345 (1.345)
C4-C5	1.433	1.436 (1.421)	1.435 (1.420)	1.433 (1.417)	1.431 (1.418)	1.425 (1.426)
C5=C6	1.364	1.349 (1.358)	1.349 (1.358)	1.341 (1.351)	1.359 (1.367)	1.354 (1.354)
N1-C6	1.357	1.354 (1.347)	1.355 (1.348)	1.351 (1.344)	1.362 (1.351)	1.357 (1.355)
C2=O/S	1.655	1.682 (1.689)	1.682 (1.696)	1.675 (1.682)	1.670 (1.684)	1.701 (1.702)
C4-N4	1.368	1.325 (1.352 *)	1.329 (1.351)	1.318 (1.346)	1.336 (1.374)	1.334 (1.332)
*Bond angles (°)*						
N-C2-N	116.3	117.8 (118.4)	117.7 (118.5)	118.0 (118.5)	117.1 (117.6)	119.6 (120.0)
C2-N3=C4	120.3	120.5 (120.2)	120.6 (120.2)	120.5 (120.1)	120.4 (119.9)	119.3 (119.0)
N3=C4-C5	123.9	121.8 (122.7)	122.0 (122.6)	121.8 (122.7)	122.4 (123.5)	122.3 (122.2)
C4-C5=C6	115.8	116.8 (116.1)	116.6 (116.1)	116.8 (116.1)	116.5 (115.7)	116.7 (116.7)
N1-C6=C5	119.7	120.0 (120.4)	120.1 (120.4)	120.1 (120.4)	119.5 (119.8)	120.1 (120.3)
C2-N1-C6	123.9	122.9 (122.1)	122.9 (122.2)	122.9 (122.1)	123.7 (123.2)	122.0 (121.7)
N1-C2=O/S	119.3	117.0 (119.0)	117.4 (119.3)	117.0 (119.0)	118.0 (120.8)	117.6 (119.1)
N3-C4-N4	116.6	118.2 (116.6)	118.3 (116.5)	118.4 (116.6)	118.1 (115.7)	118.5 (118.3)
C4-N-H4	115.2	120.3 (118.4 *)	120.1 (118.2 *)	120.2 (118.3)	119.5 (113.3)	-
C4-N-H4′	118.8	121.6 (121.7 *)	120.2 (121.5 *)	121.6 (121.8)	118.0 (117.1)	-
*Torsional angles*						
N3=C4-N-H4	14.0	0.1 (−3.6 *)	2.5 (−5.0 *)	0.3 (0.5 *)	−3.1 (−18.0)	
C5-C4-N-H4′	−23.9	0.2 (5.0 *)	−8.5 (6.7 *)	0.1 (−0.7 *)	18.1 (29.1)	
C2-N3=C4-N	176.6	−179.9 (−179.5 *)	177.6 (−179.3 *)	180.0 (179.9 *)	−178.6 (−174.4)	

* Parameters involved in H-bonds.

**Table 3:** The interaction energies E^int^(AB), deformation energies E^def^, and total CP corrected interaction energy, $\Delta E_{\mathrm{AB}}^{\mathrm{CP}}$ in kJ mol^−1^, were calculated at the B3LYP/6-311++G(3df,pd) DFT level in the tetramers and WC pairs. In the tetramer forms, AB refers to its four molecules.

Molecular System	Molecule A	Molecule B	$E_{A}^{def}$(AB)	$E_{B}^{def}$(AB)	E^def^(AB)	E^int^(AB)	$\Delta E_{AB}^{CP}$
Tetramer planar,	2TC	2TC	5.259	4.750	20.016	−210.401	−190.384
Figure 3a			5.259	4.749			
Tetramer planar,	Cytosine	Cytosine	7.716	5.053	25.535	−253.560	−228.024
Figure 3b			7.714	5.053			
Tetramer non-planar,	2TC	2TC	4.718	6.699	22.842	−221.281	−198.438
Figure 4a			4.716	6.709			
WC pairs: Figure 13a	2TC	guanine	5.774	4.780	10.554	−108.012	−97.458
Figure 14b	cytosine	guanine	5.915	5.592	11.507	−117.437	−105.930

**Table 4:** Calculated (ν^cal^, cm^−1^) and scaled wavenumbers (ν^scal^, cm^−1^) determined with the PSE procedure at the B3LYP/6-311++G(3df,pd) level in tautomers T1 and T2b of the monomer form of 2TC, together with the relative IR intensities (A, %). Comparison with the experimental wavenumbers (ν^exp^) in argon and nitrogen matrices [28] with the absolute IR intensity in parenthesis and the main characterization reported by these authors and that presented by us in the isolated state of tautomer T2b with the %PED in parenthesis. Notation: ν: stretching, β: scissoring, δ: in-plane bending, γ: out-of-plane bending, τ: torsion, and ω: wagging.

Calculated					Experimental		Characterization Ref. [28]	Characterization Present Manuscript	
T1		T2b			Ar	N_2_		Ring Mode	Assignment
ν^scal^	A	ν^cal^	ν^scal^	A	ν^exp^	ν^exp^			
3574	10	3723	3556	10	3561 (99)	3559 (129)	ν_as_(N-H) in NH_2_ (100)	33	ν_as_(N-H) in NH_2_ (100)
3451	5	3599	3443	16	3444 (152)	3441 (201)	ν_s_(N-H) in NH_2_ (99)	31	ν_s_(N-H) in NH_2_ (100)
3448	24	3199	3077	2			ν(C5-H) (91)	30	ν(C5-H) (87) + ν(C6-H) (13)
3100	0	3156	3038	4			ν(C6-H) (91)	29	ν(C6-H) (88) + ν(C5-H) (12)
3080	0	2693	2608	0	2617 (8)	2615 (23)	ν(S-H) (100)		ν(S-H) (100)
1639	100				1614 sh				
1603	14	1643	1612	100	1611 (670)	1619 (626)	β(NH_2_) (47) + ν(C=C) (13)	26	β(NH_2_) (80) + ν(C=C) (20)
		1610	1580	46	1575 (181)	1579 (206)	β(NH_2_) (35) + ν(C=C) (20)	27	ν(C=C) (33) + ν(C2-N3) (25) + ν(ring) (20) + β(NH_2_) (15)
1546	80	1590	1561	42	1562 (212)	1558 (194)	ν(C4-C5) (25) + ν(N1-C2) (20)	25	ν(C4-C5) (40) + ν(N1-C6) (27) + ν(ring) (20)
1473	54				1467 sh				
1436	8	1484	1459	21	1458 (154)	1463 (130)	δ(C5-H) (19) + ν(C-N4) (17)	24	ν(C-N) (75) + δ(C-H) (18)
1354	6	1395	1372	56	1364 (267)	1366 (215)	ν(C2-N3) (38) + δ(C6-H) (12)	21	ν(NCN) (45) + ν(CCN) (40) + δ(C-H) (12)
		1372	1350	8	1348 (154)	1350 (160)	ν(C-N4) (29) + δ(C6-H) (20)	22	δ(C6-H) (35) + ν(C-N) (30) + δ(C5-H) (22)
1296	21	1274	1255	17	1241 (166)	1245 (163)	ν(N1-C2) (29) + δ(C6-H) (29)		ν(CC, CN) (88) kekule vibration
1214	7	1246	1228	14	1216 (11)	1217 (14)	ν(N-C6) (35) + ν(C2-N3) (14)		ν(NCN, C=C) (65) + δ(C6-H) (25)
1130	27								
1098	14	1121	1106	2	1098 (9)	1100 (6)	δ(C5-H) (44) + ν(C=C) (27)	19	δ(C5-H) (70) + ν_s_(C-C-N1) (20)
1055	4	1073	1059	9	1058 (44)	1058 (46)	r(NH_2_) (49) + ν(N3-C4) (19)	18	δ_as_(NH_2_) (80)
967	4	996	983	0				16	γ(C6-H) (75) + γ(C5-H) (23)
960	0	993	980	2	979 (15)	977 (22)	δ(R1) (48) + ν(N1-C2) (12)	17	ν(ring) (95) trigonal
		954	942	4	946 (16)	941 (20)	δ(S-H) (56) + ν(C4-C5) (11)	15	δ(S-H) (30) + ν_as_(NCN) (22) + ν_as_(C-C5-C) (20)
911	2	910	899	6	891 (40)	895, 888(34)	δ(S-H) (33) + ν(C4-C5) (22)		δ(S-H) (70) + δ(C-N) (15)
775	3	819	809	8	815, 807 (47)	817, 811(47)	γ(C5-H) (52) + γ(C-N4) (35)	12	γ(C5-H) (40) + γ(C6-H) (24) + γ(C4-C5) (23)
		788	779	0				11	γ(C5-H) (60) + γ(NCN) (35)
730	2	736	727	3	724 (18)	724 (19)	τ(R1) (55) + γ(C5-H) (33)	13	δ(ring) (90)
711	0								
682	7	675	667	0	660 (4)	663,	δ(R3) (52) + ν(C4-C5) (13)		γ(C2,C4, H5) (78) + τ(NH_2_) (15)
651	2				652 (1)	660 (109)	γ(C-N4) (41) + γ(C-S) (38)		
549	0	563	556	1	556 (24)		δ(R2) (73) + ν(C-N4) (11)	8	δ(ring) (85)
537	1	492	486	4	515 (154)		τ(NH_2_) (74)	7	τ(NH_2_) (90)
466	1	441	435	0	446 (2)		τ(R3) (30) + ν(C-S) (11)	5	γ(C-N1-C) (37) + γ(ring) (30) + τ(NH_2_) (15)
433	1	436	430	1	427 (7)	446 (1)	ν(C-S) (29) + δ(ring)		γ(ring) (70) + τ(NH_2_) (25)
407	2	422	416	1	412 (4)	432 (3)	δ(C-N4) (45) + ν(C-S)	6	δ(ring) (70) + δ(NH_2_) (15) + δ(S-H) (10)
		335	329	3		413 (4)			γ(S-H) (100)
		303	297	55	307 (19)		γ(S-H) (100), ω(NH_2_) (86)	3	ω(NH_2_) (94)
264	0	237	231	1	239 (9)	300 (132)	δ(C-S) (71) + δ(C-N4) (16)	4	δ(ring) (43) + δ(C-S) (30) + δ(NH_2_) (18)
185	3	202	196	2		222 (8)		2	Puckering on N3 (80)
111	28	159	153	1					τ(ring) (88)

**Table 5:** Scaled wavenumbers (ν^scal^, cm^−1^) determined with the PSE procedure at the B3LYP/6-311++G(3df,pd) and CAM-B3LYP/6-311++G(3df,pd) levels in the tetramer forms of 2TC with tautomers T1, together with the relative IR intensities (A, %) and Raman relative intensities (S, %). Comparison with the experimental wavenumbers (ν^exp^) in the solid state [30] with the absolute IR and Raman intensities in parenthesis. The main characterization, with the ring mode number and the %PED in parenthesis.

B3LYP Non-Planar			CAM-B3LYP Planar			Experimental		Ring Mode	Assignment
ν^scal^	A	S	ν^scal^	A	S	IR	Raman		
3569, **3365**, *3353*	78	100	3587, **3420**, *3411*	40	100	3334 (82),	3334 sh,	33	ν_as_(N-H) in NH_2_ (100)
						3312 (77)	3320 (19)		
3448, ***3182***	100	32	3464, **3184**, *3182*	100	32			31	ν_s_(N-H) in NH_2_ (100)
3455, **3112**, *3110*	48	30	3473, ***3131***	22	49	3110 (90)	3109 (25)	32	ν(N1-H) (85) + ν_s_(NH_2_) (15)
***3102***, 3092	1	9	3110, *3099*	0	17	3094 (86)	3090(21),	30	ν(C5-H) (73) + ν(C6-H) (22)
							3078(19)		
3082, ***3050***	3	12	3132, ***3063***	3	19	3063 sh	3061 (11)	29	ν(C6-H) (96)
						3039 sh	3039 (11)		
1665, **1647**, 1604	50	0	1670, **1663**, *1651*	50	6		1669 (14)	26	ν(C=C) (63) + β(NH_2_) (30)
**1639**, 1633	31	2	*1645*, **1644**, 1597	4	2	1645 (93)	1630 (6)	27	β(NH_2_) (65) + ν(C=C) (35)
*1581*, **1578**	58	2	*1587*, **1583**	50	2	1580 (95)	1582 (3)	25	ν(C4-C5) (33) + δ(N1-H) (28) + ν(C-N) (23)
*1545*, **1544**	15	3	1554	14	4	1545 (80)	1551 (32)		ν(C=C) (35) + ν_as_(CN1C) (30) + β(NH_2_) (15)
						1522 (84)	1529 (24)		
*1503*, **1501**, 1494, 1490	34	2	*1509*, **1508**, 1494	22	7	1504 (82)	1495 (7)	24	ν(C4C5) (42) + ν(CN) (18) + δ(CH)(16) + β(NH_2_) (12)
**1447**, *1439*	6	1	*1459*, **1458**, 1445	4	1	1460 (50)	1463 (10)		ν_s_(C-N3-C) (72) + δ(N1-H, C6-H) (15)
**1377**, 1376, 1355	2	0	**1373**, 1352	2	0	1368 (30)	1370 (17)	22	δ(C5H, C6H) (70) + δ(NH_2_) (15) + ν(CN) (10)
1323, **1322**, *1304*, 1303	28	5	1324, 1322, ***1310***	13	10	1311(85),	1315(20),	21	ν(C2-N3) (42) + ν(C=C) (32) + δ(N1-H) (18)
						1302(100)	1301(60)		
1242	5	2	1234	4	4	1235 (75)	1249 (14)	20	δ(N1-H, C6-H) (80) + ν(N1-C6) (12)
1217			1206			1200 (64)		20	δ(N1-H) in free groups
1171, 1170, **1152**, *1150*	25	1	*1182*, **1180**, 1157	13	1	1181 (85)	1178 (5)		ν_as_(NCN) (35) + δ_as_(NH_2_) (23) + δ(C5H, N1H) (20)
						1163 sh	1167 sh		
**1109**, *1107*, 1104	5	1	*1104*, **1103**, 1100	2	2	1098 (50)	1104 (32)	19	δ(C5-H) (53) + δ_as_(NH_2_) (30) + ν(C-S) (10)
**1091**, 1090, 1057	3	0	**1083**, *1082*, 1053	1	1	1087 (59)	1092 (32)	18	δ_as_(NH_2_) (55) + δ(N1-H, C5-H) (32)
1025, 966	0	0	1030, *971*	0	3	1011 (31)	1009 (3)	16	γ(C6-H) (75) + γ(C5-H) (23)
974, **970**	1	1	***968***, 967	1	2	983 (32),	981 (28),	17	ν(ring) trigonal (95)
						967 (28)	971 (20)		
*932*, **924**	3	1	*931*, 921, **920**	2	1	940 sh	939 (13)	15	ν(ring) (92)
**902**, 644, 642	2	0	**906**, 905	1	0	930 (42)	932 (11)	10	γ(N1-H) (87)
						859 (43)	858 (1)		
**810**, 809, 756,755	2	0	**799**, 791	2	0	804 (75)	803 (4)	11	γ(C5-H) (55) + γ(C6-H) (22)+ τ(NH_2_) (12)
**765**, 760, 730,728	1	0	767, 764, 753	0	0	752 (45)	755 (3)	7	τ(NH_2_) (66) + γ(C5-H) (25)
*718*, **717**	0	3	729, 726, *717*, 716	1	6	724 (40)	718 (100)	13	δ(ring) (78) + ν(C-S) (12)
673, 671, **667**, *666*	3	0	672, 668, **665**	2	0	652 (46)	659 (5)		γ(CS) (30) + γ(CN4) (25) + γ(C5H) (18) + ω(NH_2_) (15)
**621**, *612*	5	1	638, 637, **592**, 589	3	0	94 (38)	598 (1)	3	ω(NH_2_) (84)
557, 556, ***551***, 550	0	1	553, *545*	0	2	551 (60)	550 (37)	8	δ(ring) (85)
532	0	0	530	0	0	527 (72)		7	τ(NH_2_) (80)
485, 483	0	0	*481*, **479**	1	1	482 (56)	483 (32)	6	δ(ring) (88)
462, **451**, *450*	2	2	**461**, 448	2	1	456 (54)	452 (62)		δ(ring) (78) + δ(C-S) (16)
436, 435	0	0	433, *432*	0	1	434 (32)	432 (4)		δ,γ(ring) (93)
433, **418**	1	0	428, 413	0	0	422 (27)	423 (4)		γ(N-C6-C) (57) + τ(NH_2_) (18) + γ(C6-H) (16)
294, **292**	1	0	293, 291	0	1	306 (28)	306 (15)		δ(ring) (92)
269	1	0	271, 269	0	0	289 ms	293 (21)		δ(ring) (85)
						225 vs	230 (1)		
205, **203**, 193, 189	2	0	201, 198, 181, 178	0	0	214 ms	215 (2)		γ(ring) (75) + γ(C-S) (25)
152, 151	11	0	134, 132, 127, **125**	9	0	142 vs	142 (27)		ω(NH_2_) (100)

## Data Availability

Data are contained within the article.
